# m6A genotypes and prognostic signature for assessing the prognosis of patients with acute myeloid leukemia

**DOI:** 10.1186/s12920-023-01629-1

**Published:** 2023-08-18

**Authors:** Caizhu Fu, Ruirui Kou, Jie Meng, Duanfeng Jiang, Ruilan Zhong, Min Dong

**Affiliations:** Hematology, the Second Affiliated Hospital of Hainan Medical University, Haikou, 570000 China

**Keywords:** TCGA, Acute myeloid leukemia, N6-methyladenosine, Prognostic, Immune

## Abstract

**Background:**

N6-methyladenosine (m6A) has been confirmed to function critically in acute myeloid leukemia (AML) progression. Hitherto, the subtyping and prognostic predictive significance of m6A-correlated genes in AML is unclear.

**Method:**

From The Cancer Genome Atlas (TCGA-LAML), Therapeutically Applicable Research to Generate Effective Treatments (TARGET-AML) and Gene Expression Omnibus (GEO, GSE71014) databases, we collected the sequencing data of AML patients. The batch effect was removed via limma package for TCGA-LAML and TARGET-AML, and the aggregated samples were AML cohorts. Samples in the AML cohort identified m6A models in AML by consensus clustering based on 23-m6A-related modulators. M6A-related differentially expressed genes (m6ARDEGs) influencing the overall survival (OS) of AML were determined by performing differential expression analysis and univariate COX analysis, and consensus-based clustering was utilized to access AML molecular subtypes. LASSO and multivariate COX analyses were performed to obtain the optimized m6ARDEGs to construct the m6A Prognostic Risk Score (m6APR_Score). Whether the model was robust was evaluated according to Kaplan–Meier (K-M) and receiver operator characteristic (ROC) curves. Further, the abundance of immune cell infiltration was explored in different m6A modification patterns and molecular subtypes and m6APR_Score groupings. Finally, nomogram was constructed to predict OS in AML. Quantitative real-time polymerase chain reaction (RT-qPCR) and cell counting kit-8 (CCK-8) assay were used to validate the genes in m6APR_Score in AML cells.

**Results:**

The m6A models (m6AM1, m6AM2, m6AM3) and molecular subtypes (C1, C2, C3) were identified in the AML cohort, exhibiting different prognosis and immunoreactivity. We recognized novel prognostic biomarkers of AML such as CD83, NRIP1, ACSL1, METTL7B, OGT, and C4orf48. AML patients were grouped into high-m6APR_Score and low-m6APR_Score groups, with the later group showing a better prognosis than former one. Both the AML cohort and the validation cohort GSE71014 demonstrated excellent prediction. Finally, the nomogram accurately predicted the survival of patients suffering from AML. Further, the decision curves showed that both nomogram and m6APR_Score showed excellent prediction. It was confirmed in vitro experiments that mRNA expressions of NRIP1, ACSL1, METTL7B and OGT were elevated, while CD83 and C4orf48 mRNA expressions downregulated in AML cells. A significant increase in the viability of U937 and THP-1 cell lines after inhibition of CD83, while siMETTL7B had contrast results.

**Conclusion:**

Our study demonstrated that m6APR_Score and CD83, NRIP1, ACSL1, METTL7B, OGT, and C4orf48 potentially provided novel and promising prognostic support for AML patients.

**Supplementary Information:**

The online version contains supplementary material available at 10.1186/s12920-023-01629-1.

## Introduction

Acute myeloid leukemia (AML) refers to a type of scarce blood disorder of adult origin in the bone marrow, and the incidence of AML known with age predisposition, with a progressive increase in incidence with age [1]. Although numerous researchers had reached consensus through extensive studies, risk stratification guidelines for predicting the prognosis of AML patients were published [2]. However, as a highly aggressive malignant tumor, AML recurred in the majority of patients even after taking hematopoietic stem cell transplantation treatment (HSCT), and according to statistical data, the 5-year survival rate of AML was only 28.3% [1, 3]. Benefiting from the rapid development of sequencing technologies, several studies carried out recently reported novel recognized prognostic markers in AML at the molecular scale [4, 5]. These studies revealed the molecular landscape of AML at the proteomic and genomic scales and made advances in the direction of improving AML prognosis. However, AML, a lethal nonsolid tumor, demonstrated extreme tumor heterogeneity across patients, which also resulted in the inability to accurately assess prognosis in clinical operations [6]. Therefore, exploring to uncover effective novel prognostic diagnostic markers in AML is crucial to improve patient prognosis as well as quality of life.

N6-methyladenosine (m6A) is the commonest form of RNA methylation modification in cells in the molecular level, and aberrant m6A modification directly affected the central law process [7, 8]. Accumulated m6A was carcinogenic and triggered the development of several cancers [9, 10]. For example, m6A was shown to be intimately associated with the pathogenesis of breast cancer [11], colorectal cancer [12], and glioma [13]. Not coincidentally, m6A modifications also regulated AML production. sheng and colleagues reported that the m6A-related gene YTHDC1 influenced the proliferative effects of AML cells, and furthermore, that abnormally high expression of YTHDC1 promoted AML cell appreciation and inhibited the spontaneous renewal process of leukemic stem cells through the MCM complex [14]. Yankova and colleagues also confirmed that the m6A methyltransferase METTL3 represented a critical gene in AML disease development [15]. Thus, systematic insight into the potential mechanisms of m6A in AML holds promise for identifying effective therapeutic and prognostic biomarkers.

Considering the potential worth of m6A in AML, we identified different m6A models in AML as well as molecular subtypes. Differentially expressed genes affected by m6A were further established, and then prognostic prediction models and nomogram were constructed as m6A signatures. In addition, this study also explored the different m6A models as well as immune cell infiltration differences in molecular subtypes, which further extended the clinical value of m6A-related prognostic signatures in AML.

## Materials and methods

### Dataset acquisition and pre-processing

In this study, the RNA-Seq dataset (TPM, transcripts per million) TCGA-LAML of AML was uploaded through TCGA (https://www.cancer.gov/about-nci/organization/ccg/research/structural-genomics/tcga) database, and 132 primary tumor samples were maintained after screening. The expression files TARGET-AML 156 of AML samples (TPM, transcripts per million) were acquired through TARGET (https://ocg.cancer.gov/programs/target) database. To minimize the experimental error, the TCGA-LAML and TARGET data were processed to remove the batch effect using the removeBatchEffect function of the limma package ([16]), and the processed samples were named as AML cohort. The sequencing file GSE71014 was downloaded from the GEO (https://www.ncbi.nlm.nih.gov/geo/) database for 104 AML patients. we used the AML cohort as the training set and GSE71014 as the validation set.

### Consensus clustering for identification of m6A models

Formula clustering analysis using the “ConsensusClusterPlus” R package (Version3.17) with km algorithm and 1-Spearman correlation was performed to identify m6A models of samples in the AML cohort based on the 23-m6A modulators included 8 writers (ZC3H13, RBM15B, RBM15, VIRMA, WTAP, METTL16, METTL14, METTL3), 11 readers (HNRNPC, FMR1, YTHDC1, YTHDC2, YTHDF1, YTHDF2, YTHDF3, LRPPRC, IGF2BP3, IGF2BP2, IGF2BP1, RBMX, HNRNPA2B1), and 2 erasers (FTO, ALKBH5) [17] in an earlier study with reference to the method of Wilkerson et al. [18].

### Identification of Differentially Expressed Genes (DEGs) and m6A subtypes

DEGs in different m6A models (|log2FC|> 1 & FDR < 0.05) were identified using the limma package, which we defined as m6A-related DEGs (m6ARDEGs). Univariate COX analysis was performed on m6ARDEGs to obtain m6ARDEGs that could influence AML prognosis (*p* < 0.01). Based on the expression data of these m6ARDEGs, consensus clustering analysis was performed on samples from the AML cohort to identify m6A-associated molecular subtypes in AML.

### Prognostic assessment model construction and validation

LASSO and multivariate COX regression analyses were conducted on m6ARDEGs capable of influencing AML prognosis to construct an AML prognostic model [19], and the AML prognostic risk score (m6APR_Score) was calculated by the following equation.

$$m6APR\_Score=\sum{coef}_i\ast Exp\;{gene}_i$$

where coefi refered to the prognosis-related m6ARDEGs expression level and coefi was the COX regression coefficient of the corresponding prognosis-related m6ARDEGs. According to the median value, the samples were classified into high-m6APR_Score group and low-m6APR_Score group. An AML mRNA expression, from Oregon Health & Science University (OHSU), was downloaded from cBioPortal for Cancer Genomics (https://www.cbioportal.org/) acted as external validation dataset (OHSC-AML dataset).

Differences in OS of patients from different m6APR_Score subgroups were assessed by K-M analysis. The prognostic predictive performance of m6APR_Score was evaluated using ROC curve. Finally, whether the model was robust was validated in the validation set GSE71014.

### Assessment of the abundance of immune cell infiltration

The relative abundance of 22 immune cell species in tumor tissue was quantified using the CIBERSORT algorithm (https://cibersort.stanford.edu/) [20]. The scores of 10 immune cell species were analyzed using the MCP-Count function [21].

### GSVA enrichment analysis

For different m6A models and m6A subtypes, h.all.v7.5.1.symbols.gmt was downloaded from the HALLMARK database to obtain the biological signaling pathways contained therein [22], and the signaling pathway differences in different m6A models and m6A subtypes were calculated by the GSVA package [23].

### Construction of nomogram and decision curve

Univariate and multivariate COX analyses were performed combining m6APR_Score, clinical traits of AML samples to identify significant independent factors for AML prognosis. Subsequently, nomogram predicting AML 1-year, 3-year and 5-year survival risk were generated using the rms package by combining m6APR_Score and prognostically significant independent factors, and the prediction accuracy of the nomogram was analyzed using calibration curves (https://rdrr.io/cran/rms/). Finally, the robustness of m6APR_Score and nomogram in predicting AML prognosis was evaluated by drawing decision curves.

### Cell culture and transient transfection

GM12878 (BNCC360167), THP-1 (BNCC358410) and U937 (BNCC359322) cells were purchased from Beijing Bena Biotechnology Co. (Beijing, China). Cells were cultured in DEME F-12 medium. Transfection of the negative control (NC), CD83 siRNA and METTL7B siRNA (Sagon, China) was conducted by applying Lipofectamine 2000 (Invitrogen, USA). GGGGCAAAATGGTTCTTTCGACG (CD83-si) and ACCCAAATCCCCACTTTGAGAAG (METTL7B-si) were the target sequences for CD83 siRNA and METTL7B siRNA.

### RT-qPCR

The total RNA from GM12878, THP-1 and U937 cell lines (Thermo Fisher, USA) was extracted using TRIzol reagent. cDNA was created from 500 ng of RNA using the HiScript II SuperMix (Vazyme, China). By applying the SYBR Green Master Mix, RT-qPCR was carried out in ABI 7500 System (Thermo Fisher, USA). 45 cycles of 94 °C for 10 min, 94 °C for 10 s, and 60 °C for 45 s each comprised the PCR amplification conditions. Table 1 displayed the list of the sequences of primer pairs for targeted genes.

**Table 1:** The primers of genes

Genes	Forward primer sequence (5’-3’)	Reverse primer sequence (5’-3’)
CD83	AAGGGGCAAAATGGTTCTTTCG	GCACCTGTATGTCCCCGAG
NRIP1	GGATCAGGTACTGCCGTTGAC	CTGGACCATTACTTTGACAGGTG
ACSL1	CCATGAGCTGTTCCGGTATTT	CCGAAGCCCATAAGCGTGTT
METTL7B	GCAACCGCAAGATGGAGAG	GATTTGGGTCTAGGCAGGTGA
OGT	TCCTGATTTGTACTGTGTTCGC	AAGCTACTGCAAAGTTCGGTT
C4orf48	CGTCCGAATGGGCGTTTTC	TGCATGAACTCGAAGGCGT
GAPDH	AATGGGCAGCCGTTAGGAAA	GCCCAATACGACCAAATCAGAG

### Cell viability

The CCK-8 (Beyotime, China) was conducted to measure cell viability. In 96-well plates, cells from various treatments were grown at a density of 1 × 103 cells per well. Solution CCK-8 was used. Using a microplate reader, the OD 450 values of each well were determined during a 2-h incubation at 37 °C (BioTeK, USA).

### Statistical analysis

The software packages utilized in this study were obtained from R software (https://www.r-project.org/, R version 4.2.2). Data pre-processing via Sangerbox platform (http://www.sangerbox.com/, [24]. T-test and kruskal.test were tested to assess statistical differences between groups and *p* < 0.05 was considered statistically significant in this study.

## Results

### Distinct m6A model in AML

**Fig. 1:**
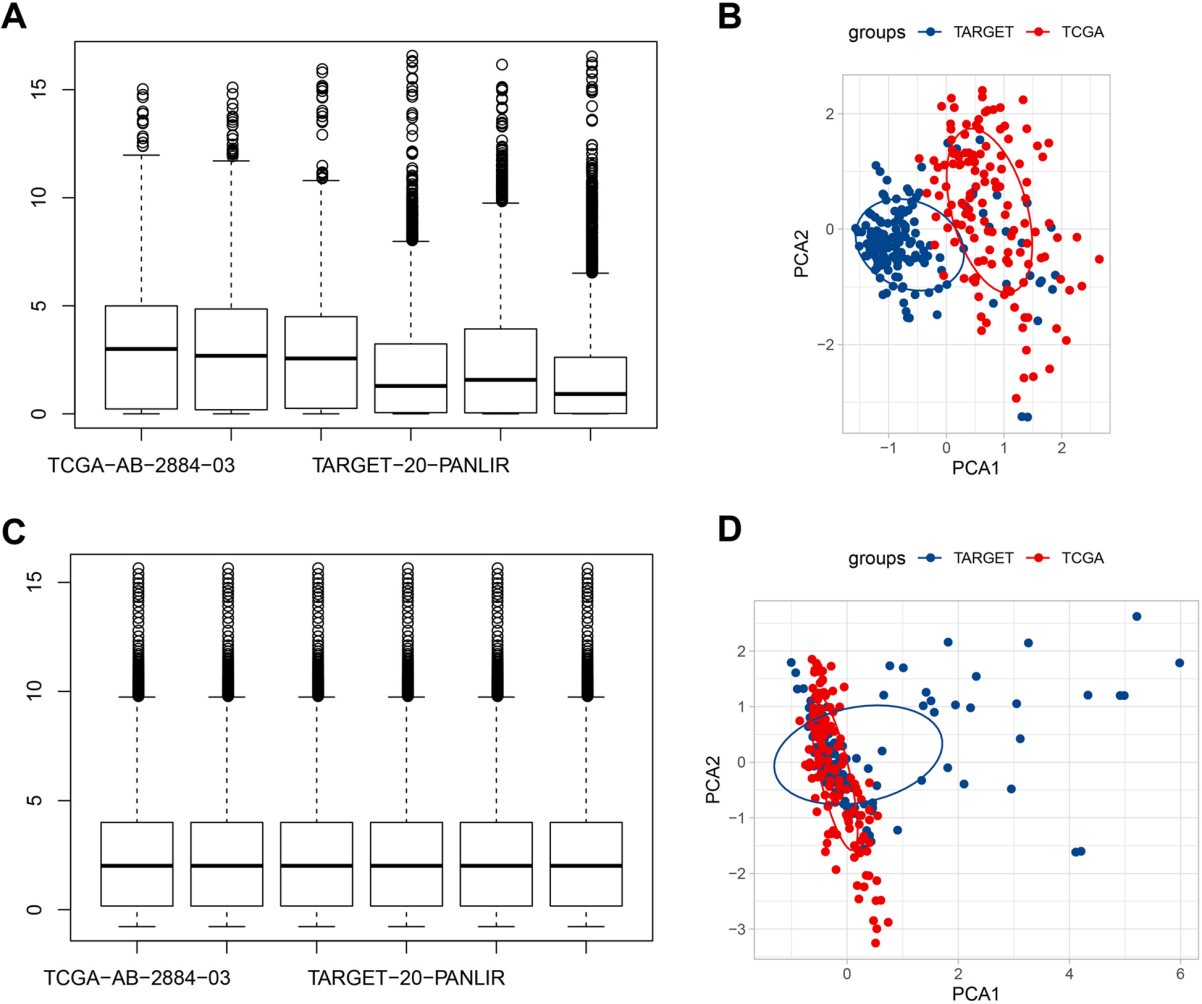
TCGA-LAML and TARGET-AML data pre-processing. **A**-**B** TCGA-LAML and TARGET-AML gene expression boxplots and PCA results (**C**-**D**) Boxplots of TCGA-LAML and TARGET-AML gene expression after removal of batch effects and PCA results

**Fig. 2:**
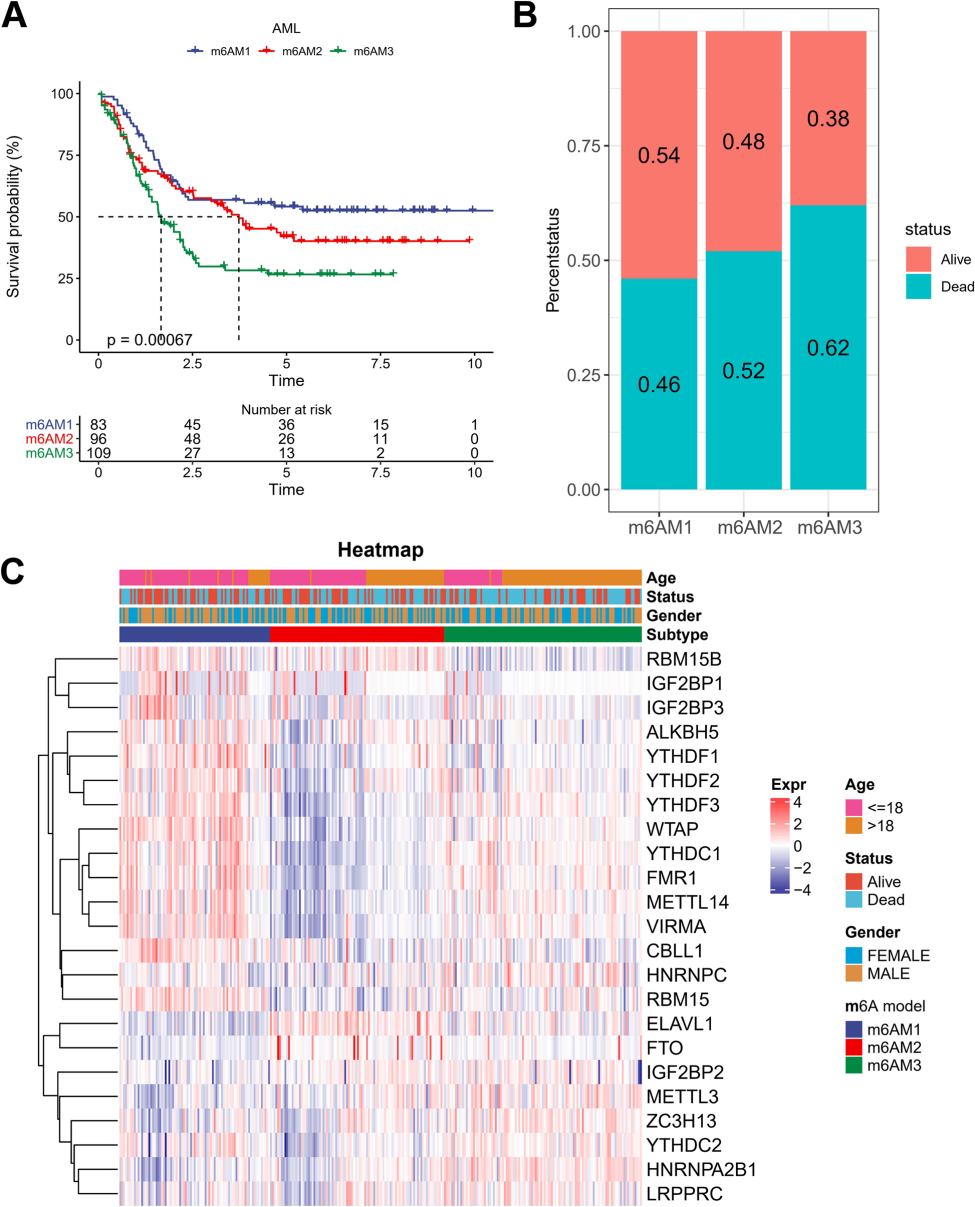
Identification of m6A models in the AML cohort. **A** K-M curves for the three m6A models of the AML cohort (**B**) Statistical bar graph of the difference in survival status between the three m6A modification patterns (**C**) Heat map of differences in 23-m6A-related modulators, survival status and clinical information among the three m6A models

Initially, the TCGA and TARGET data sets were merged and named the AML cohort. To reduce experimental error, the batch effect of samples in the AML cohort was eliminated using the removeBatchEffect function in the limma package. Eventually, totally 288 samples from the AML cohort were included for subsequent analysis (Fig. 1A-D). Next, based on the 23- m6A-related modulators, the 288 samples in the AML cohort could be grouped into three m6A models containing m6A model 1 (m6AM1), m6A model 2 (m6AM2), and m6A model 3 (m6AM3) (Supplementary Fig. 1A-C). K-M survival curves for m6AM1-3 showed that the m6AM1 had best prognosis, followed by the m6AM2, and the m6AM3 had poorest prognosis (Fig. 2A). Survival status statistics showed that the highest proportion of cases died in the m6AM3 (Fig. 2B). Eventually, the heatmap presented information on 23-m6A-related modulators expression levels, survival status, age, and gender for 288 AML samples in m6AM1-3 (Fig. 2C). Most m6ARGs expressions were higher in m6AM1 and m6AM3 than those in m6AM2.

### Tumor microenvironment (TME) difference and Gene Set Variation Analysis among three m6A models

**Fig. 3:**
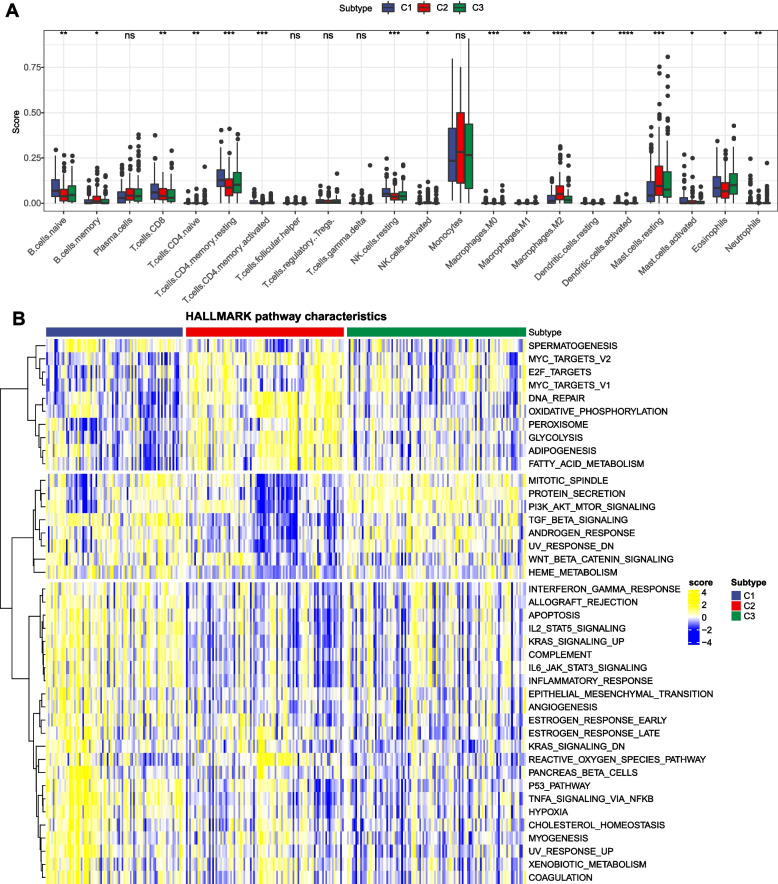
TME differences between different m6A models. **A** 22-Relative abundance of immune cells (**B**) Differences in biological pathway activity. ns *p* > 0.05; * *p* < 0.05; ** *p* < 0.01; ***p < 0.001; **** *p* < 0.0001

For the purpose of elucidating the TME situation in m6AM1-3, the CIBERSORT algorithm was used to analyze the relative abundance of immune cells in TME, and it was clearly observed that the abundance of B cells naive, T.cells.CD8, T cells CD4 memory resting, and NK cells resting were higher in m6AM1, Mast.cells.resting, B.cells.memory, Macrophages.M2 were higher in m6AM1, while Eosinophils was higher in m6AM3 (Fig. 3A). Next, single-sample gene set enrichment analysis (ssGSEA) was performed to explore the biological pathway differences in m6AM1-3. We noticed that APOPTOSIS, P53 PATHWAY were activated in m6AM1; DNA REPAIR, OXIDATIVE PHOSPHORYLATION, ADIPOGENESIS were activated in m6AM2; cancer related pathways as PI3K AKT MTOR SIGNALING, WNT-β CATENIN SIGNALING in m6AM3 were activated (Fig. 3B). From these discoveries it was evident that m6AM1 was mainly associated with apoptosis as well as the cell cycle, m6AM2 was mainly associated with cellular energy metabolic pathways, and cancer-related pathways were activated in m6AM3. This also accounted for the survival differences among the three m6A models.

### Identification of m6A-related DEGs (m6ARDEGs) and m6A subtypes

**Fig. 4:**
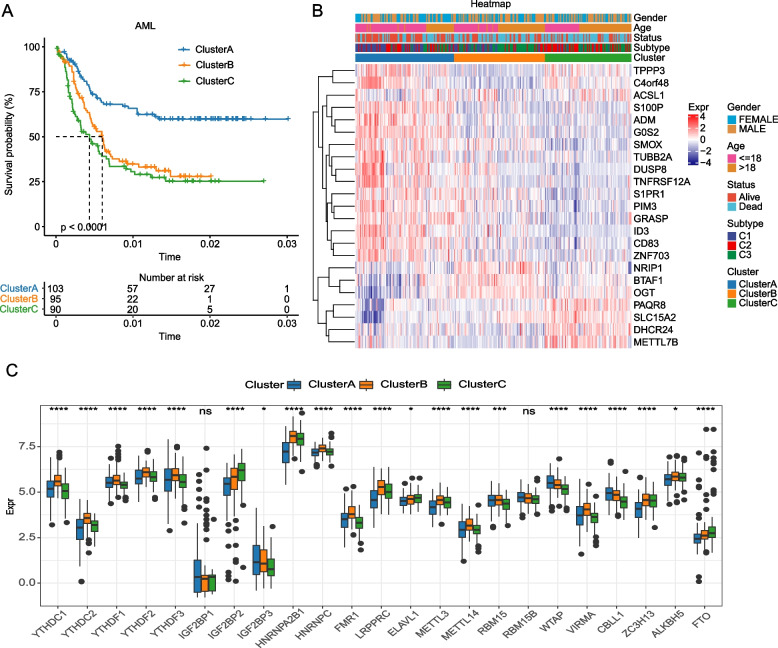
Identification of molecular subtypes of AML. **A** K-M survival curves for the three molecular subtypes in the AML cohort (**B**) Heatmap of 23-m6ADEGs, survival status, and clinical information in the three molecular subtypes (**C**) Box plot of 23-m6ADEGs expression

To further investigate the gene-transcription level differences in the three m6A models, limma package was used to mine the m6ARDEGs in m6AM1 vs m6AM2_m6AM3 (m6AM1 group), m6AM2 vs m6AM1_m6AM3 (m6AM2 group) and m6AM3 vs m6AM1_m6AM2 (m6AM3 group). Totally 131 m6ARDEGs were identified in m6AM1-3 (FDR < 0.05 & |log2FC|> 1). Univariate COX screened 23 m6ARDEGs that were significantly and significantly associated with OS in AML (*p* < 0.01). The unsupervised clustering algorithm was performed to cluster the samples in the AML cohort into three subtypes (m6A cluster A, m6A cluster B, m6A cluster C) (Supplementary Fig. 2A-C). K-M survival curves showed significantly different OS for m6A cluster A-C (*p* < 0.0001) (Fig. 4A). The expression of 23-m6ARDEGs in m6A cluster A-C were illustrated in Fig. 4B, C.

### TME and enrichment difference

**Fig. 5:**
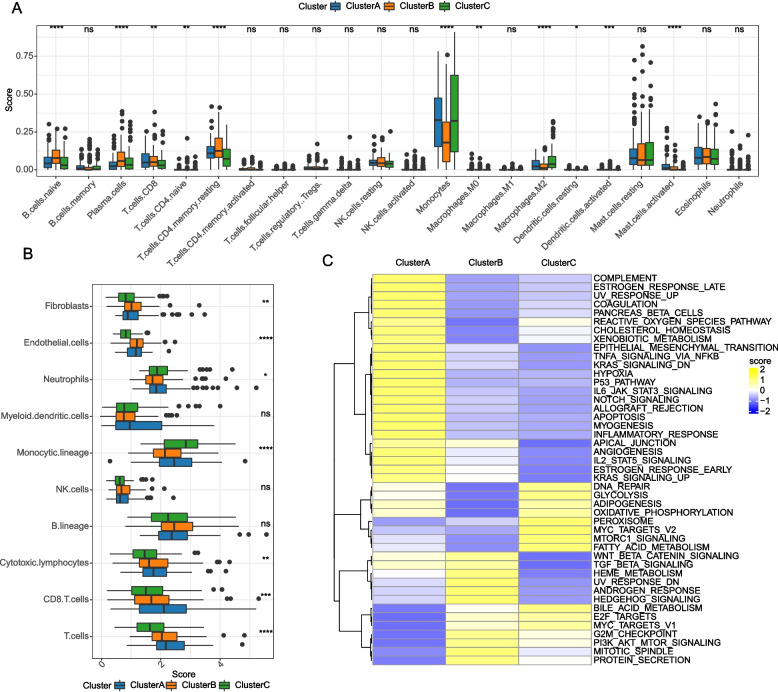
Immunological/pathway activity among molecular subtypes. **A** Relative abundance of 22 immune cells (**B**) Infiltration score of 10 immune cells (**C**) Differences in biological pathway activity

To explore TME differences among cluster A-C, CIBERSORT and MCP-count were utilized to assess the level of immune cell infiltration. From the results of CIBERSORT analysis, it was observed that Monocytes, Mast cells activated were richer in cluster A (*p* < 0.0001). CD4 memory resting, Plasma cells, B cells naive, T cells, T.cells.CD8 were richer in cluster B (*p* < 0.0001) (Fig. 5A). MCP-count the command output was as follows, there were significantly differences in immune cell scores of Fibroblasts, Endothelial cells, Cytotoxic lymphocytes, Neutrophils, CD8 T cells, Monocytic lineage, T cells among cluster A-C, most of which in cluster C were lower than cluster A and cluster B (Fig. 5B). In inclusion, it was clearly evident that TNFA SIGNALING VIA NFKB, KRAS SIGNALING UP, P53 PATHWAY were significantly activated in cluster A; WNT BETA CATENIN SIGNALING pathway, MITOTIC SPINDLE, PI3K AKT MTOR SIGNALING showed significant activation in cluster B; GLYCOLYSIS, ADIPOGENESIS, OXIDATIVE PHOSPHORYLATION, FATTY ACID METABOLISM pathway were significantly activated in cluster C (Fig. 5C).

### Establishment and validation of the m6A Prognostic Risk Score (m6APR_Score)

**Fig. 6:**
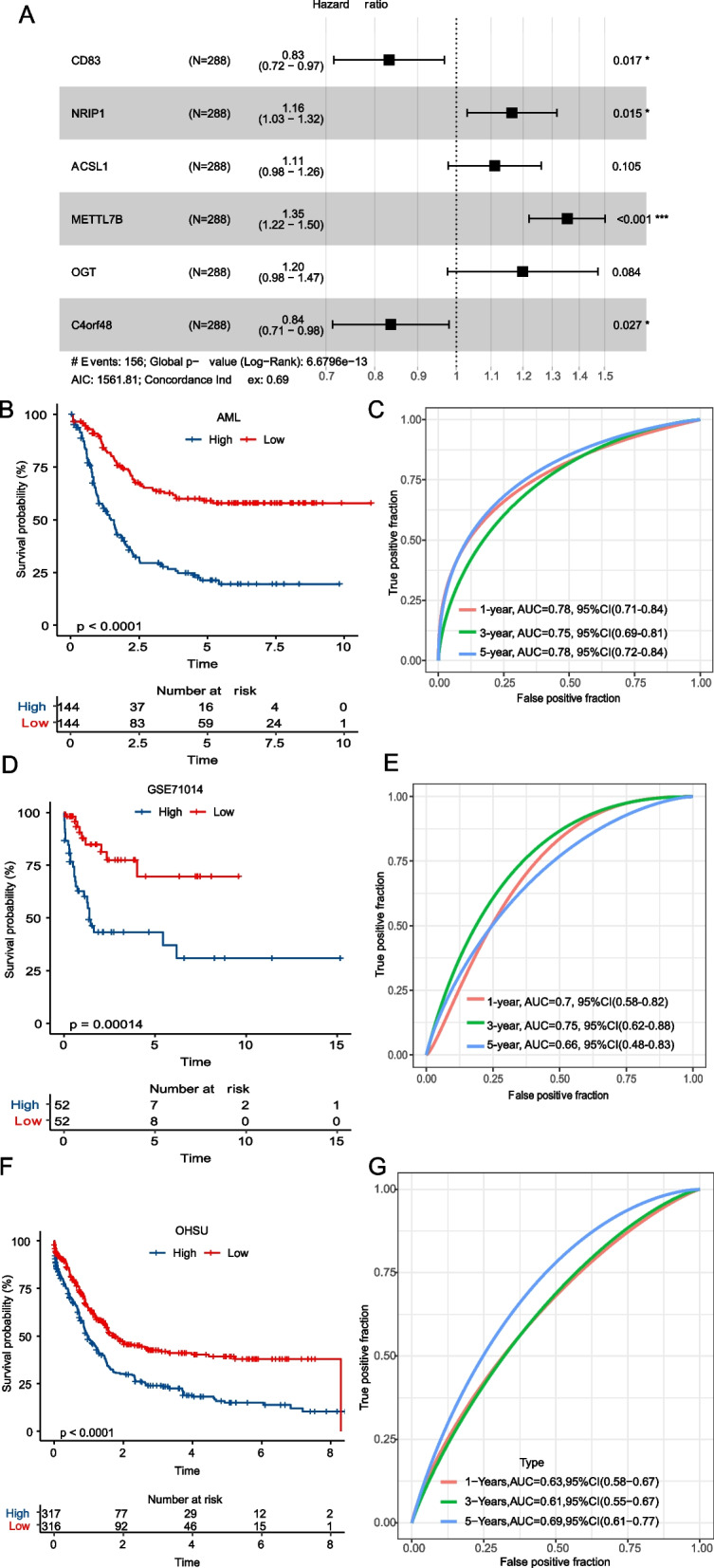
Prognostic risk stratification model construction for AML. **A** Forest plot of 6-m6ARDEGs (**B**-**C**) K-M survival curves and ROC curves of patients in different m6APR_Score groups of the AML cohort (**D**-**E**) K-M survival curves and ROC curves of patients in different m6APR_Score groups of GSE7101

The prognostic model assessed via m6APR_Score was established in the AML cohort based on 23-m6ARDEGs. LASSO and multivariate Cox regression were determined to dig out optimal prognostic m6ARDEGs, overall, CD83, NRIP1, ACSL1, METTL7B, OGT and C4orf48 were dug out as optimal prognostic m6ARDEGs (Fig. 6A). Meanwhile, m6APR_Score was defined: m6APR_Score = -0.184*CD83 + 0.152*NRIP1 + 0.105*ACSL1 + 0.304*METTL7B + 0.182*OGT-0.179*C4orf48. 288 samples were contributed to high-m6APR_Score group and low-m6APR_Score group. Moreover, Kaplan–Meier analysis in two m6APR_Score of AML cohorts revealed that low-m6APR_Score AML patients demonstrated positive prognosis (Fig. 6B). the area under ROC curve (AUC) of 1-, 3-, and 5-years were all over than 0.7, which were 0.78, 0.75 and 0.78 respectively (Fig. 6C). Then, the similar analysis was conducted to verified the predictive robustness of m6APR_Score in GSE71014 cohort. There was a clear observation of a significant difference in prognosis between high- and low-m6APR_Score patients, with high m6APR_Score having better OS and showing better AUC values at 1-, 3- and 5-years (Fig. 6D, E). In OHSC-AML dataset, an obviously prognosis differences between high- and low- group was observed, and a well AUC also presented (Fig. 6F, G).

### Construction of nomogram and decision curve

**Fig. 7:**
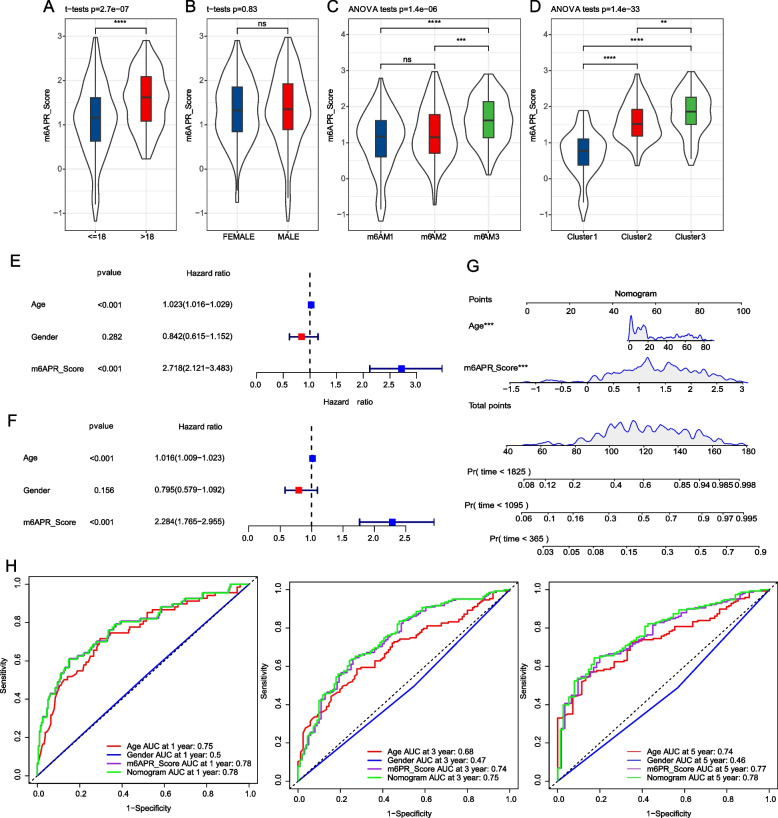
m6APR_Score independence analysis and Nomogram construction. **A**-**D** Differences in m6APR_Score in age, gender, m6A models and m6A clusters (**E**–**F**) Univariate and multivariate COX regression analysis of m6APR_Score combined with age, gender (**G**) Nomogram of m6APR_Score combined with age (**H)** The ROC curves of a variety of clinical features for overall survival (OS) at 1, 3 and 5 years

To examine m6APR_Score differences in AML subgroups, we counted m6APR_Score in age, Gender, m6A model and m6A cluster subgroups. We observed that older AML patients had higher m6APR_Score (Fig. 7A). Conversely, there was no significant correlation between m6APR_Score and gender (Fig. 7B). Simultaneously, we also found higher m6APR_Score in m6AM3 and C3 patients, which explained the worse prognosis of m6AM3 and C3 (Fig. 7C, D). Univariate and multivariate Cox analysis of m6APR_Score and clinical features showed m6APR_Score, age as independent prognostic factors (Fig. 7E, F). To quantify the prognostic risk of AML patients, we combined the m6APR_Score and age to construct nomogram as in Fig. 7G. We further assessed the model's predictive power using the calibration curve, and we found that the projected calibration curves for the 1-, 3-, and 5-year calibration points almost exactly matched the standard curve, demonstrating the nomogram's outstanding predictive power (Supplementary Fig. 3A). Meanwhile, ROC curves showed that nomogram and m6APR_Score provided highest sensitivity and specificity in predicting AML patient's OS compared to clinical features (Fig. 7H). Additionally, decision curve revealed that both m6APR_Score and Nomogram benefits were significantly greater than the extreme curves (Supplementary Fig. 1C).

### Evaluation of immune signature in different m6APR_Score groups

**Fig. 8:**
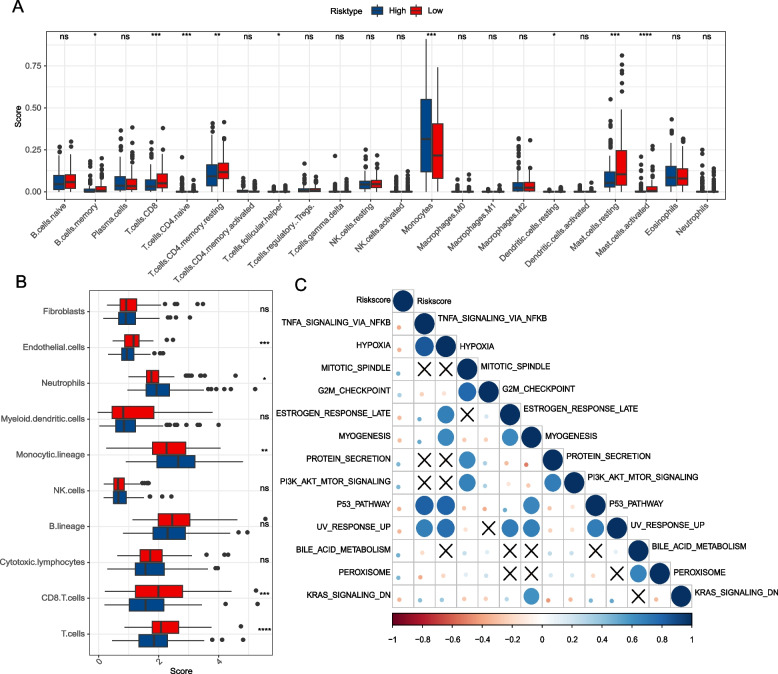
Differences in the immunological/biological pathway activity of different m6APR_Score groups. **A** Relative abundance of 22 immune cells (**B**) Immune infiltration score of 10 immune cells (**C**) Heat map of m6APR_Score correlation with cancer-related pathways. ns *p* > 0.05; * *p* < 0.05; ** *p* < 0.01; *** *p* < 0.001; **** *p* < 0.0001

Twenty two immune cell abundance were evaluated by CIBERSORT method between two groups, we found the abundance of T.cells.CD8, T cells CD4 memory resting, Mast cells resting, Mast cells activated were Significant higher in low-m6APR_Score group (Fig. 8A). The results of the MCP-count analysis were aligned highly with the CIBERSORT. Higher immune infiltration of T cells CD8, T cells and Endothelial cells were in the m6APR_Score group (Fig. 8B). Then, the potential correlation between m6APR_Score and the HALLMARK pathway illustrated by heatmap (Fig. 8C), intuitively, m6APR_Score showed significant positive relation to HYPOXIA, P53 PATHWAY, MYOGENESIS, ESTROGEN RESPONSE LATE, TNFA SIGNALING VIA NFKB, KRAS SIGNALING DN, UV RESPONSE UP pathways.

### Experimental verification of the expression and function of m6APR_Score model genes

**Fig. 9:**
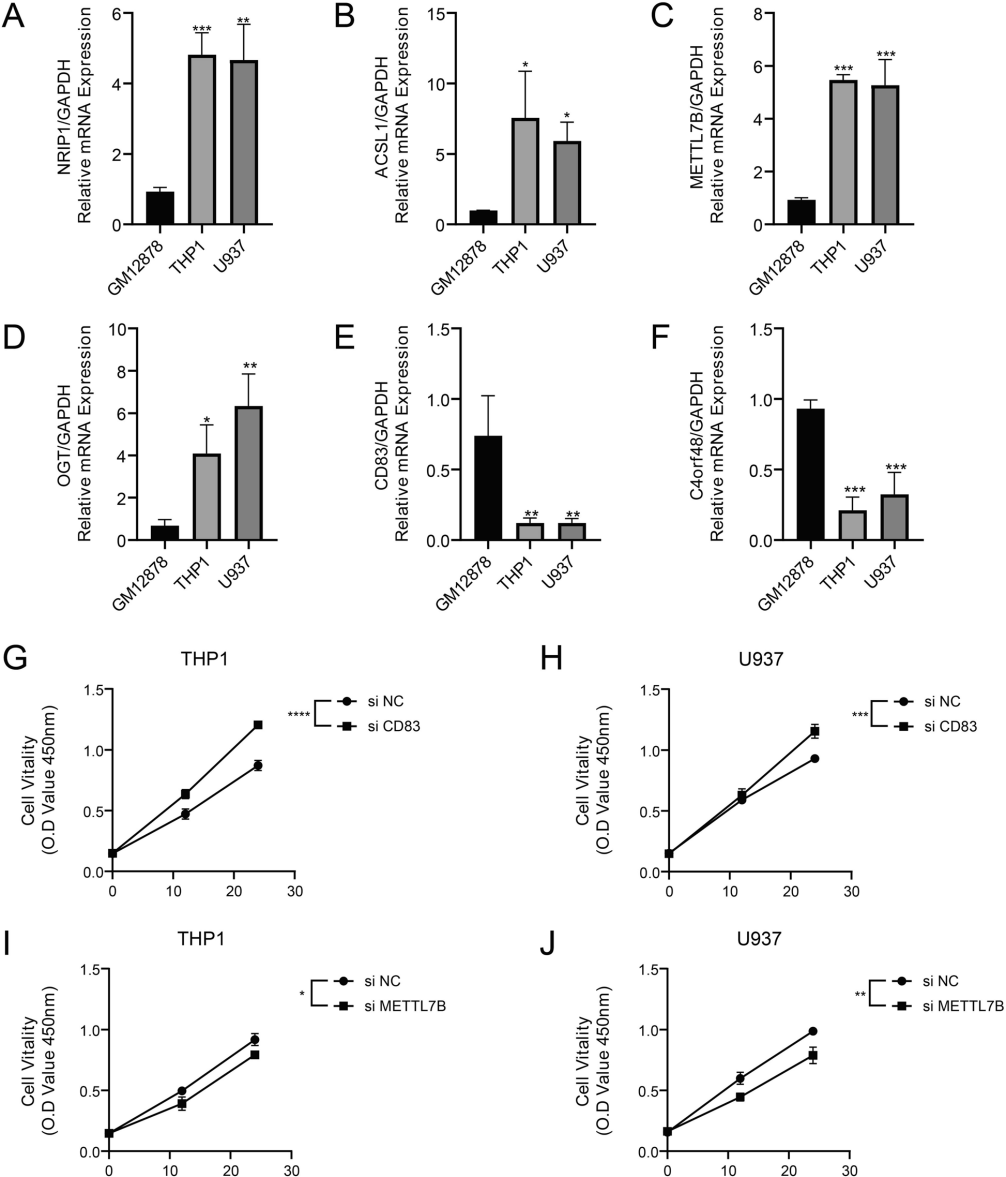
Experimental verification of the expression and function of m6APR_Score model genes. **A**-**F** Results of RT-qPCR assays for NRIP1, ACSL1, METTL7B, OGT, CD83 and C4ORF48 in GM12878, THP-1 and U937 cell lines (*n* = 3). **G**-**H** CCK8 assay results of THP-1 and U937 cell lines after CD83 inhibition (*n* = 3). **I**-**J** CCK8 assay results of THP-1 and U937 cell lines after METTL7B inhibition (*n* = 3). * ≤ 0.05, ** ≤ 0.01, *** ≤ 0.001, **** ≤ 0.0001. The results are presented as mean ± SD

To test the expression and function of the bioinformatics model, we detected the mRNA expression of CD83, NRIP1, ACSL1, METTL7B, OGT, C4orf48 by RT-qPCR in GM12878, THP1 and U937 cell lines. The expressions of NRIP1, ACSL1, METTL7B and OGT could be observed to be elevated in THP-1 and U937 compared to normal hematopoietic cell GM12878 (Fig. 9A-D). While the expression of CD83 and C4orf48 were significantly downregulated in U937 cell and THP-1 cell lines (Fig. 9E, F). Subsequently, based on risk score assignment, we verified the viability of THP-1 and U937 cell lines after interference with CD83 and METTL7B. The results showed a significant increase in the viability of U937 cell and THP-1 cell lines after inhibition of CD83 (Fig. 9G, H). In contrast, the viability of U937 cell and THP-1 lines decreased significantly after inhibition of METTL7B (Fig. 9I, J). These data also validated the validity of the risk score model.

## Discussion

In recent years, the contribution of m6A methylation modification of mRNAs in the development of malignant tumors became an emerging research topic in the medical field [25]. Studies revealed that m6A influenced and altered gene expression mainly by interfering with and regulating mRNA splicing, translation and stability [26]. A report suggested that long-term cumulative m6A modifications disrupted the balance between value addition and differentiation of normal cellular haematopoietic stem cells (HSG), and that pivotal regulators in m6A had potential therapeutic value for disease [27, 28].

Benefiting from the development and down-streaming of high-throughput sequencing technologies, a variety of molecular subtypes were defined at the molecular level based on the expression levels of critical disease genes that differ from traditional clinicopathological histology. These new molecular subtypes complemented traditional pathological histological subtypes and provided an active role in the study of disease mechanisms and innovative therapeutic tools, as well as clarification of the molecular biology of cancer [29, 30]. Review of current published studies revealed that there were relatively few studies related to different m6A models in AML. In this study, based on the expression data of m6ARGs, there were three different m6A models existed in AML patients. Among them, the m6AM2 was distinctly associated with the cell cycle and the m6AM3 was distinctly associated with cancer progression. Notably, m6AM2 and m6AM3 clearly showed poor prognosis. In addition, m6AM2 and m6AM3 evidently demonstrated higher levels of FTO and IGF2BP2 expression. Our study demonstrated high consistency with the results of Xu et al. [18]. Therefore, the findings of this study further confirmed the different m6A models and prognostic differences in AML. In-depth studies on the molecular basis of the occurrence and development of these molecular subtypes and the search for new targets for intervention would have positive consequences in the direction of developing effective new anti-cancer drugs and improving patients' clinical prognosis.

In this study, we screened 6-m6ARDEGs as novel prognostic biomarkers, containing CD83, NRIP1, ACSL1, METTL7B, OGT, and C4orf48. The CD83-targeted chimeric antigen receptor (CAR) T cell targeted therapy was proposed based on the report by Shrestha and colleagues that CD83 was a novel target against graft-versus-host disease in AML patients undergoing bone marrow hematopoietic stem cell transplantation [31]. Grasedieck and colleagues proposed that NRIP1 expression in AML bone marrow tissues was up-regulated by the regulation of the AML marker oncogene EVI1, which was unique to patients with abnormal chromosome 3 [32]. Existing studies shown that upregulation of ACSL1 is regulated by SNHG7/ miR-449a to cause proliferation and migration of thyroid cancer cells. SNHG7 adsorbed miR-449a and competitively reduced the interaction between miR-449a and ACSL1 [33]. METTL7B was essential for cancer cell proliferation and tumorigenesis in non-small cell lung cancer (NSCLC), and METTL7B is a promising therapeutic target for NSCLC [34]. Asthana and colleagues found in a mouse subcutaneous xenograft model of AML that inhibition of OGT function led to the differentiation and apoptosis of AML cells and the remission of cancer symptoms [35]. However, C4orf48 was a newly discovered biomarker of AML, and its function in other types of tumors remained unclear. In addition, it was found that the function of NRIP1 and METTL7B genes was regulated by m6A modification [36, 37]. In this study, we also found that mRNA expressions of NRIP1, ACSL1, METTL7B and OGT were elevated, while CD83 and C4orf48 mRNA expressions downregulated in AML cells. a significant increase in the viability of THP-1 and U937 cell lines after inhibition of CD83, while siMETTL7B had contrast results. Above findings indicated the Rationality and feasibility of m6APR_Score.

Our research showed that the three m6A clusters, three m6A gene clusters, and m6Ascore were strongly linked with the relative abundance of 22 immune cell infiltration. It is now understood that the m6A alteration is crucial for controlling the immunological response [38]. B-cells, CD8 + T-cells, CD4 + T-cells, neutrophils, macrophages, and neutrophil expression of METTL7B correlated positively with each other, but negatively with dendritic cells [39]. Expression levels of T Cell CD8 and T Cell CD4 naive were higher in the low- m6APR_Score group. Previous reports indicated that T Cell CD8 and T Cell CD4 function affected clinical outcomes in cancer patients [40, 41]. Patients at high- m6APR_Score group had significantly poor prognosis and we hypothesized that T Cell CD8 and T Cell CD4 were activated in patients at high prognostic risk and that immune checkpoints derived from T cells might trigger immune escape of tumor cells by blocking immune cell function.

Overall, we ascertained the different m6A models in AML based on the 23-m6A modulators. Finally, a 6-gene prognostic model was created, and the model was highly robust. However, there remained shortcomings in this study. Firstly, this study was conducted using data from earlier studies in public databases for sublicensing, and there was a lack of clinical cohorts to validate the robustness of the model. Second, although we ascertained 6 m6A-associated prognostic genes, we did not further explore the specific biomolecular mechanisms of these genes in relation to m6A modification in AML in both in vivo and in vitro assays. Further investigation of the relationship between these genes and AML and m6A modification is necessary.

## Conclusion

Our study constructed a 6-gene prognostic predictive model for predicting prognosis in AML, which constructed an m6A signature that could also serve as an independent prognostic factor for AML compared to traditional clinicopathological prognostic factors. We also preliminarily identified different m6A models in AML and their association with the immune landscape. This study provided new scientific evidence for effective and accurate clinical prediction of AML prognosis, and m6A modification patterns in AML might also help medical research practitioners to better understand AML disease mechanisms.

### Supplementary Information

**Additional file 1: Supplementary Figure 1**. Identification of m6A models. (A) CDF curves in consensus clustering (B) Clustering consistency at k = 2-10 (C) Heatmap of sample consistency for optimal clustering groupings.

**Additional file 2: Supplementary Figure 2**. Identification of the m6A molecular subtypes. (A) CDF curves in consensus clustering (B) Clustering consistency at k = 2-10 (C) Heatmap of sample consistency for optimal clustering groupings.

**Additional file 3: Supplementary Figure 3**. m6APR_Score independence analysis and evaluation of Nomogram predictive performance. (A) 1-year, 3-year, 5-year calibration curves for Nomogram (B) Decision curves for Nomogram, m6APR_Score.

**Additional file 4.**

**Additional file 5.**

## Data Availability

The datasets generated during and analyzed during the current study are available from the corresponding author on reasonable request.

## Abbreviations

AML: N6-methyladenosine
AUC: Area under ROC curve
CDF: Cumulative distribution function
FC: Fold change
GEO: Gene Expression Omnibus
HR: Hazard ratio
K-M: Kaplan–Meier
LASSO: Least absolute shrinkage and selection operator
m6A: N6-methyladenosine
m6ARGs: M6A-related genes
m6ARDEGs: M6A-related differentially expressed genes
m6APR_Score: M6A Prognostic Risk Score
OS: Overall survival
ROC: Receiver operating characteristic analysis
ssGSEA: Single-sample gene set enrichment analysis
TCGA: The Cancer Genome Atlas
TARGET: Therapeutically Applicable Research to Generate Effective Treatments
TME: Tumor microenvironment
