# Supplementary figures and images for: m6A genotypes and prognostic signature for assessing the prognosis of patients with acute myeloid leukemia
Source: BMC Med Genomics. 2023 Aug 18;16:191. doi: 10.1186/s12920-023-01629-1 (PMC10436408; doi:10.1186/s12920-023-01629-1)

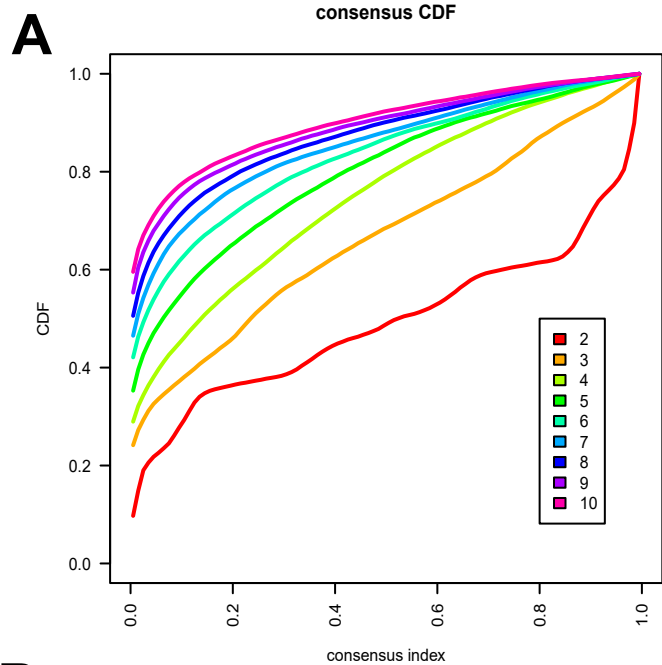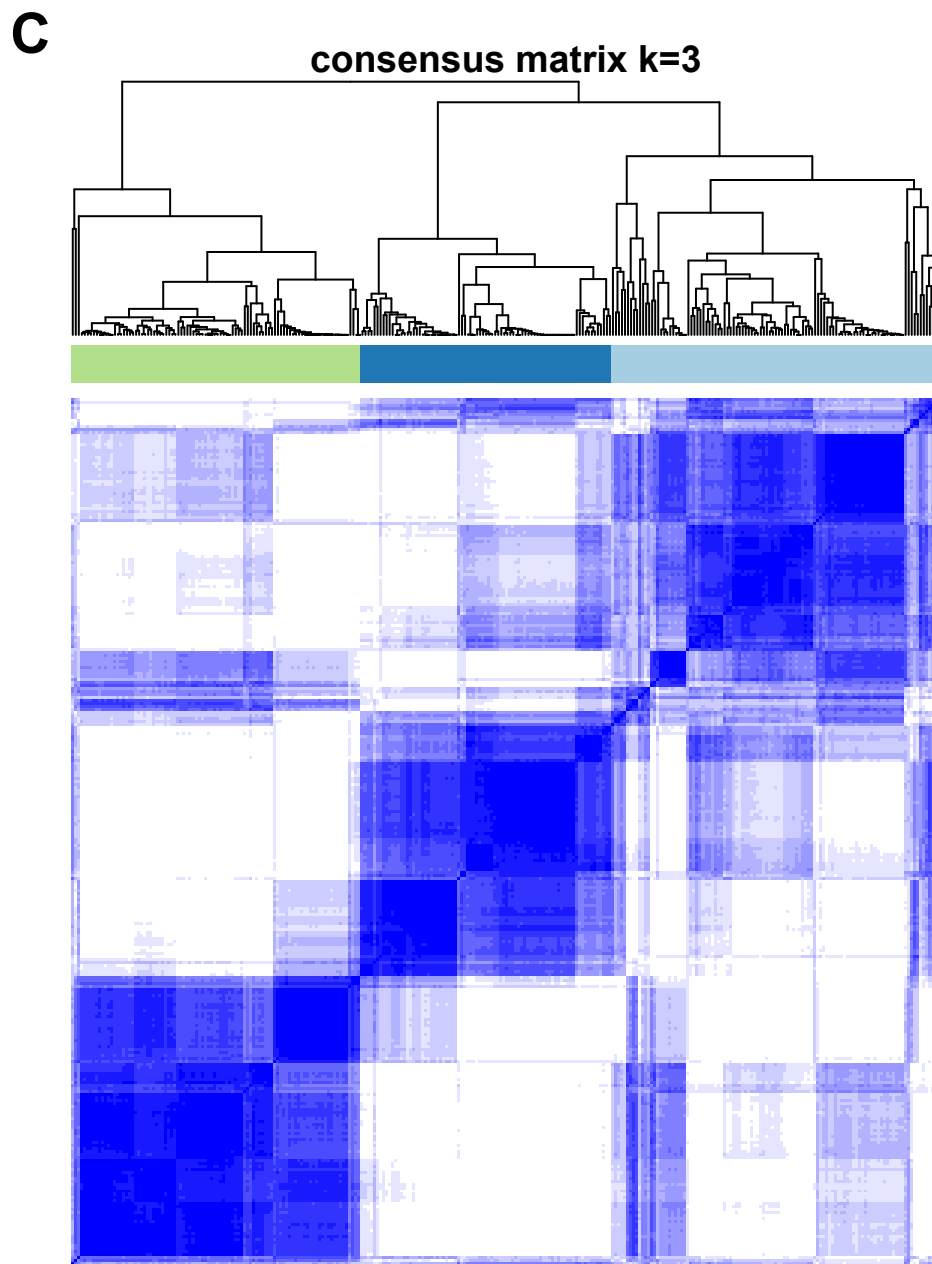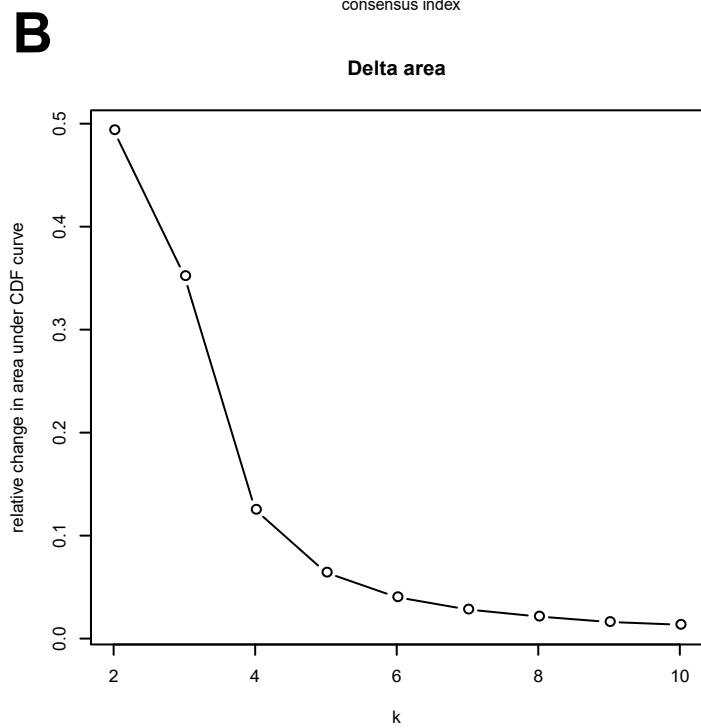

Supplement: Supplementary file 1 — Additional file 1: Supplementary Figure 1. Identification of m6A models. (A) CDF curves in consensus clustering (B) Clustering consistency at k = 2-10 (C) Heatmap of sample consistency for optimal clustering groupings. [file 12920_2023_1629_MOESM1_ESM.pdf]

**A**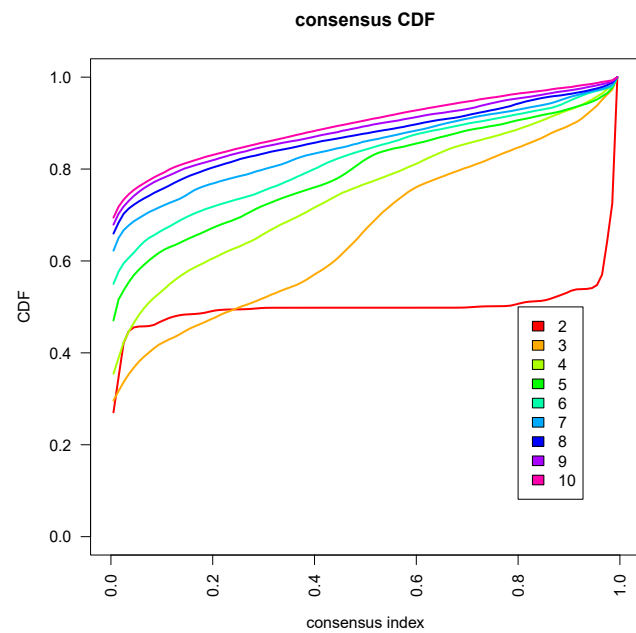**B**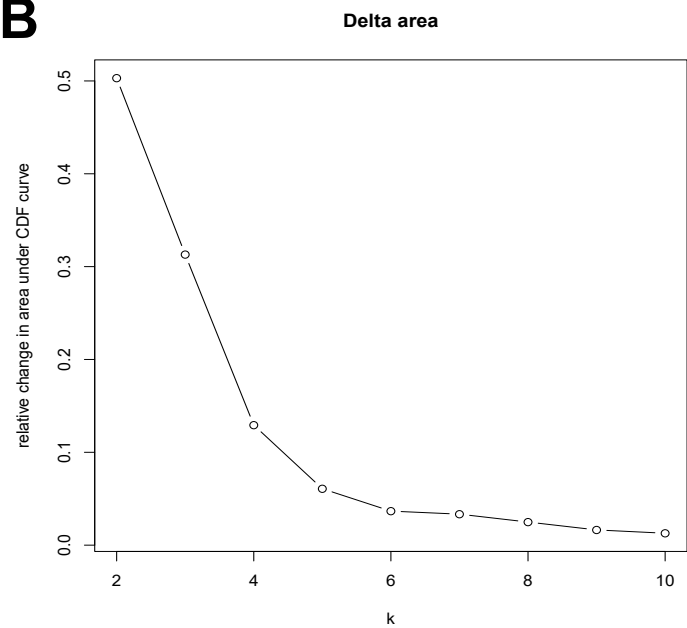**C**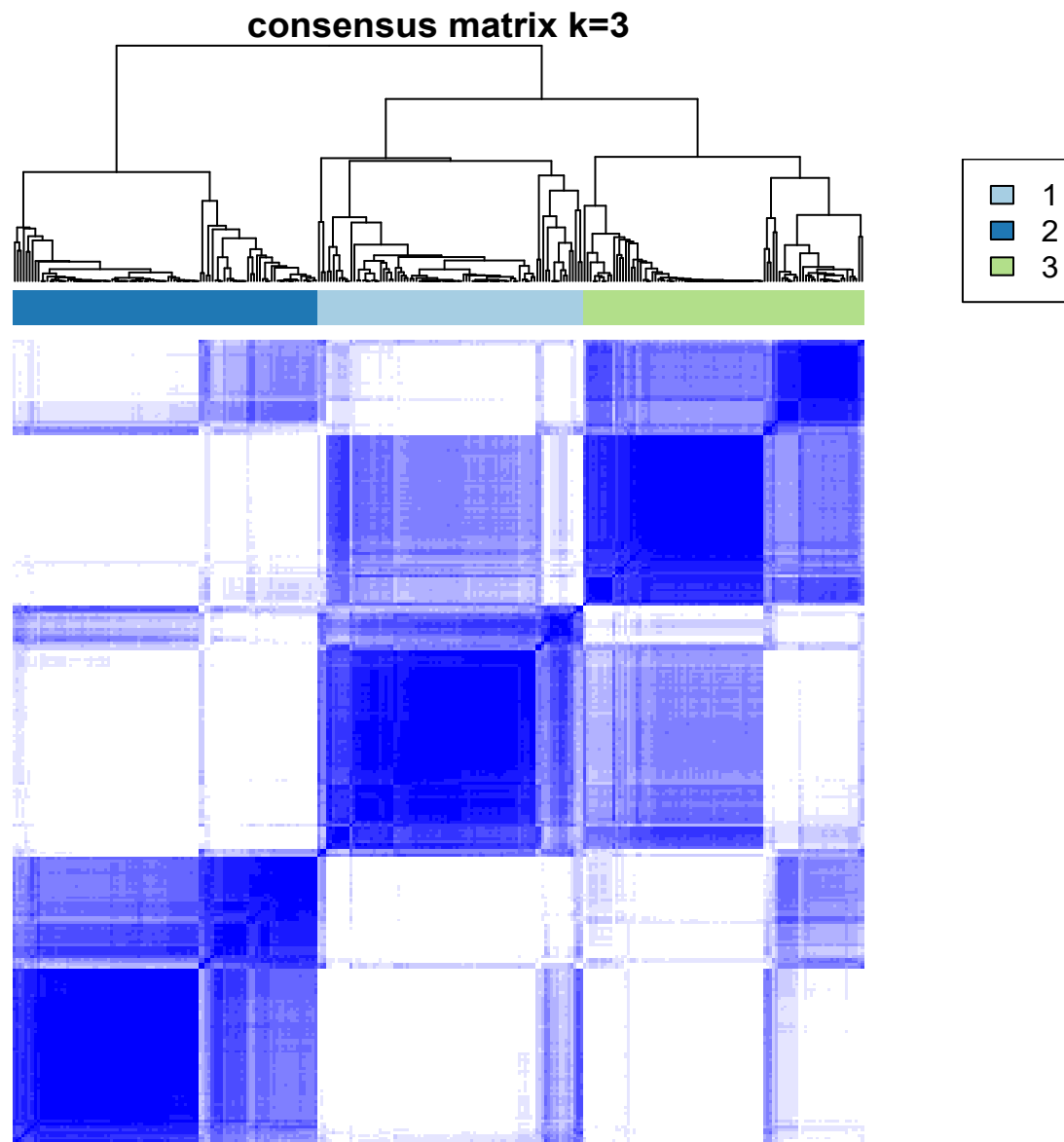

Supplement: Supplementary file 2 — Additional file 2: Supplementary Figure 2. Identification of the m6A molecular subtypes. (A) CDF curves in consensus clustering (B) Clustering consistency at k = 2-10 (C) Heatmap of sample consistency for optimal clustering groupings. [file 12920_2023_1629_MOESM2_ESM.pdf]

**A**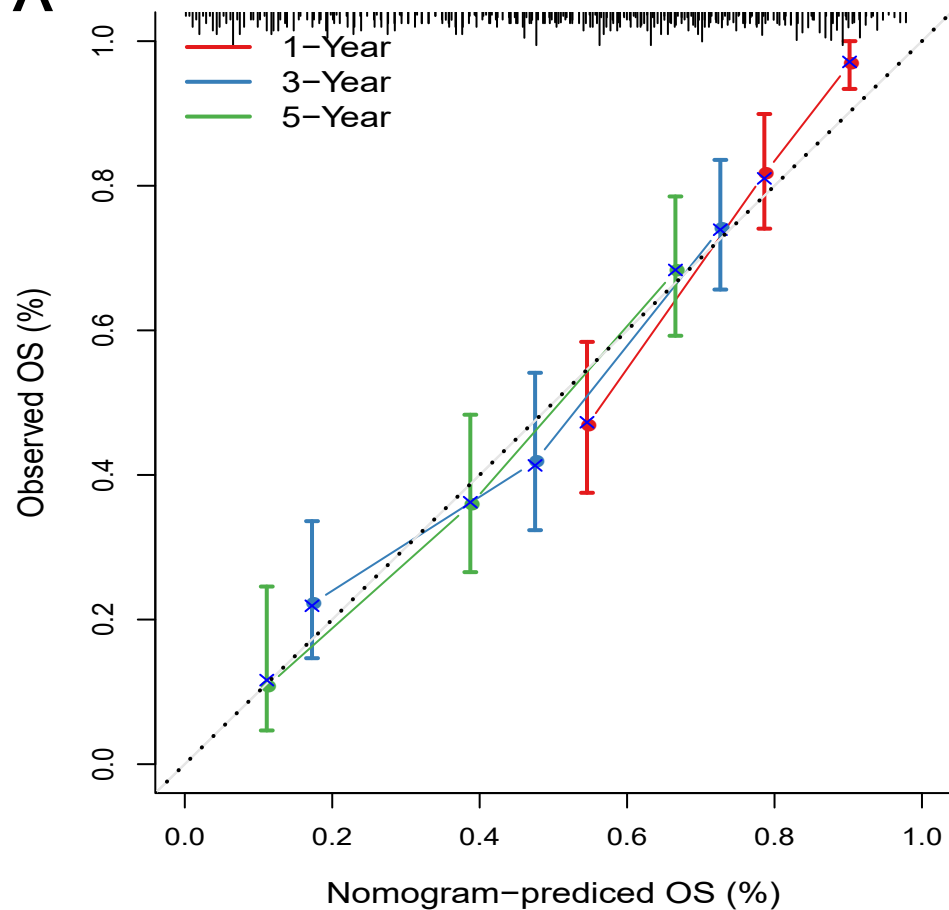**B**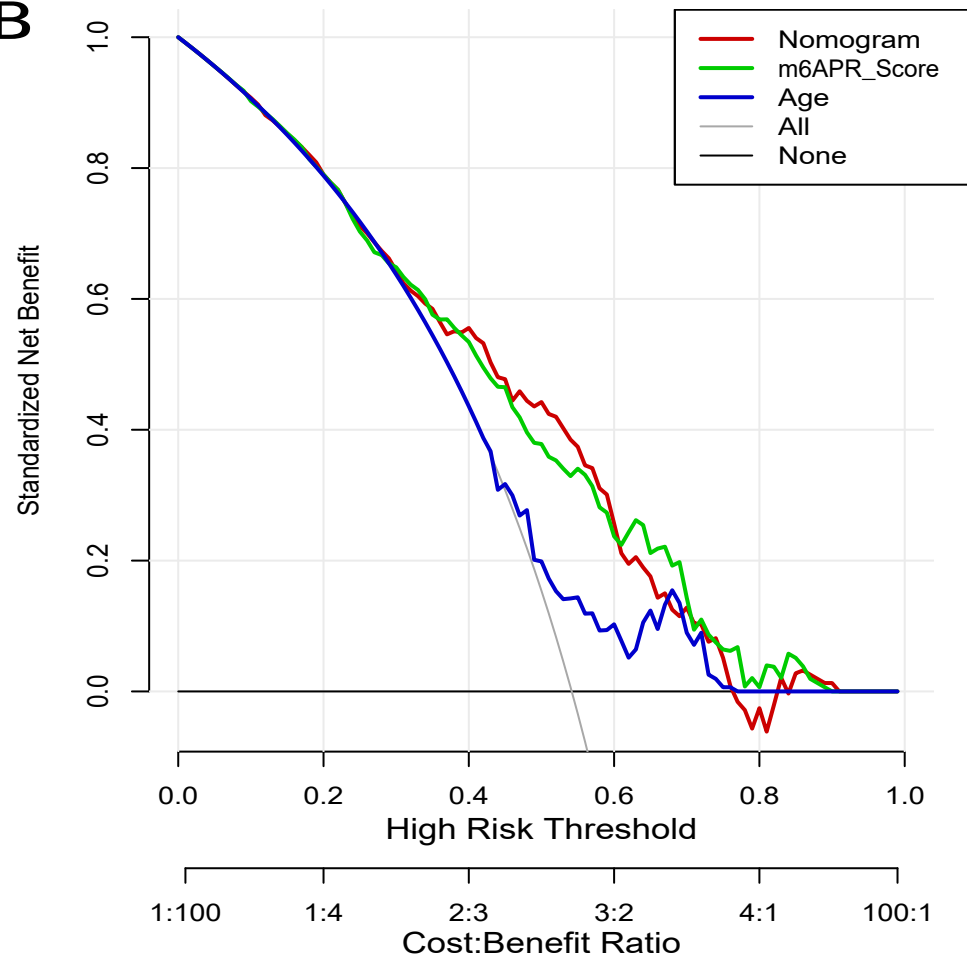

Supplement: Supplementary file 3 — Additional file 3: Supplementary Figure 3. m6APR_Score independence analysis and evaluation of Nomogram predictive performance. (A) 1-year, 3-year, 5-year calibration curves for Nomogram (B) Decision curves for Nomogram, m6APR_Score. [file 12920_2023_1629_MOESM3_ESM.pdf]
