# Supplementary material for: m6A genotypes and prognostic signature for assessing the prognosis of patients with acute myeloid leukemia
Source: BMC Med Genomics. 2023 Aug 18;16:191. doi: 10.1186/s12920-023-01629-1 (PMC10436408; doi:10.1186/s12920-023-01629-1)
Supplement: Supplementary file 4 — Additional file 4. [file 12920_2023_1629_MOESM4_ESM.pdf]

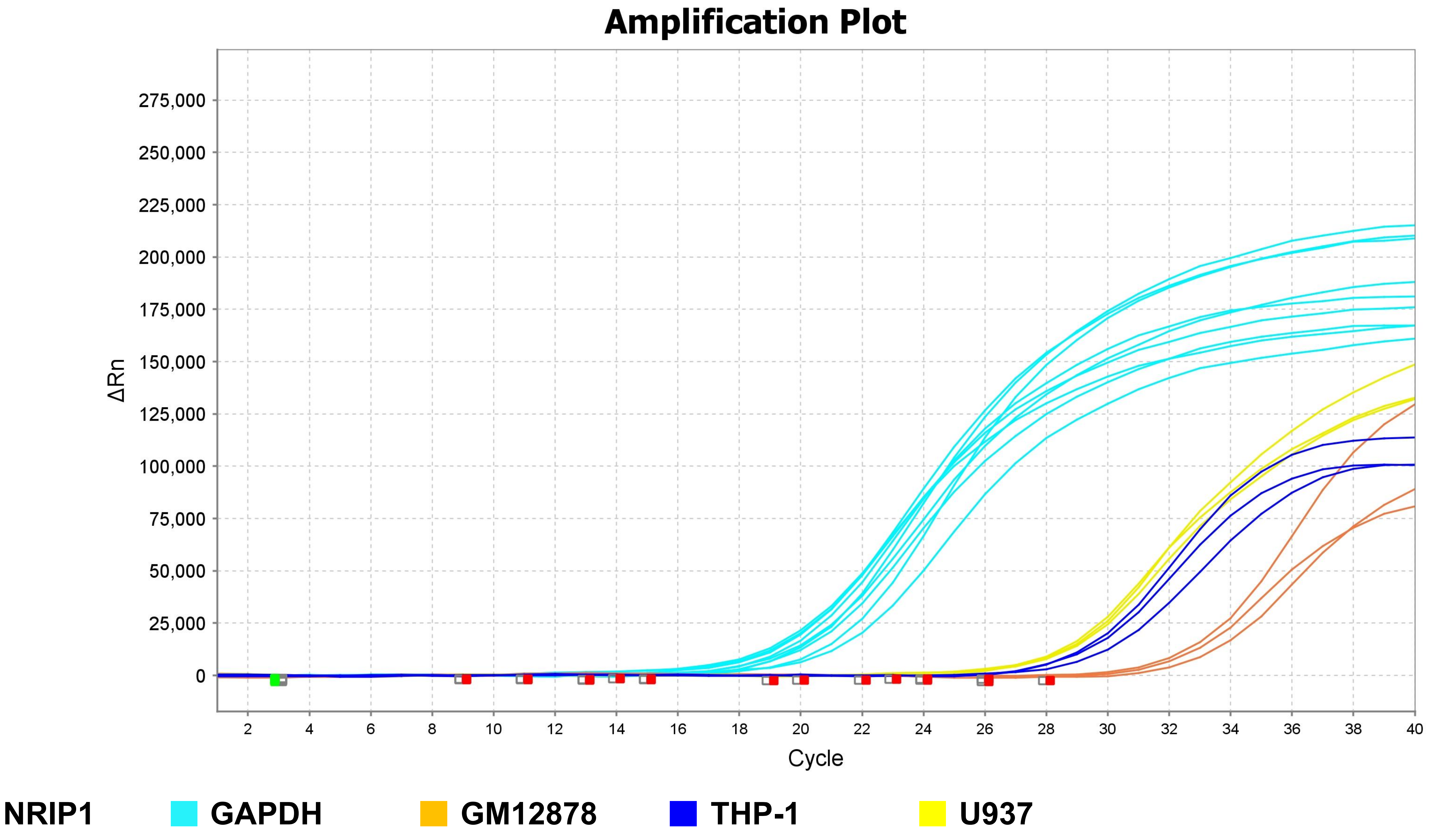
Amplification Plot
ΔRn
275,000
250,000
225,000
200,000
175,000
150,000
125,000
100,000
75,000
50,000
25,000
0
2
4
6
8
10
12
14
16
18
20
22
24
26
28
30
32
34
36
38
40
Cycle
NRIP1
GAPDH
GM12878
THP-1
U937

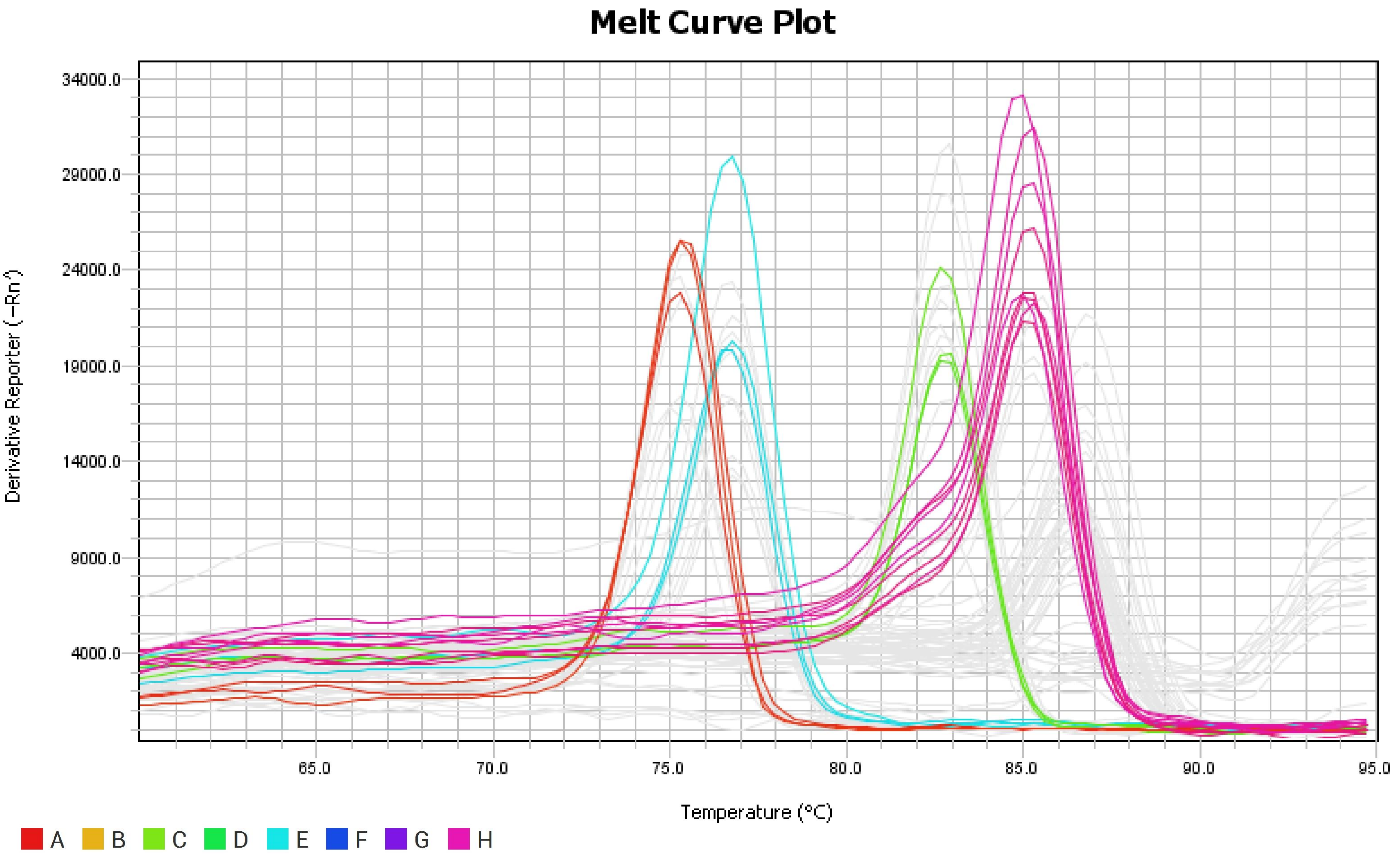
Melt Curve Plot
Derivative Reporter (−Rn′)
34000.0
29000.0
24000.0
19000.0
14000.0
9000.0
4000.0
65.0
70.0
75.0
80.0
85.0
90.0
95.0
Temperature (°C)
A
B
C
D
E
F
G
H

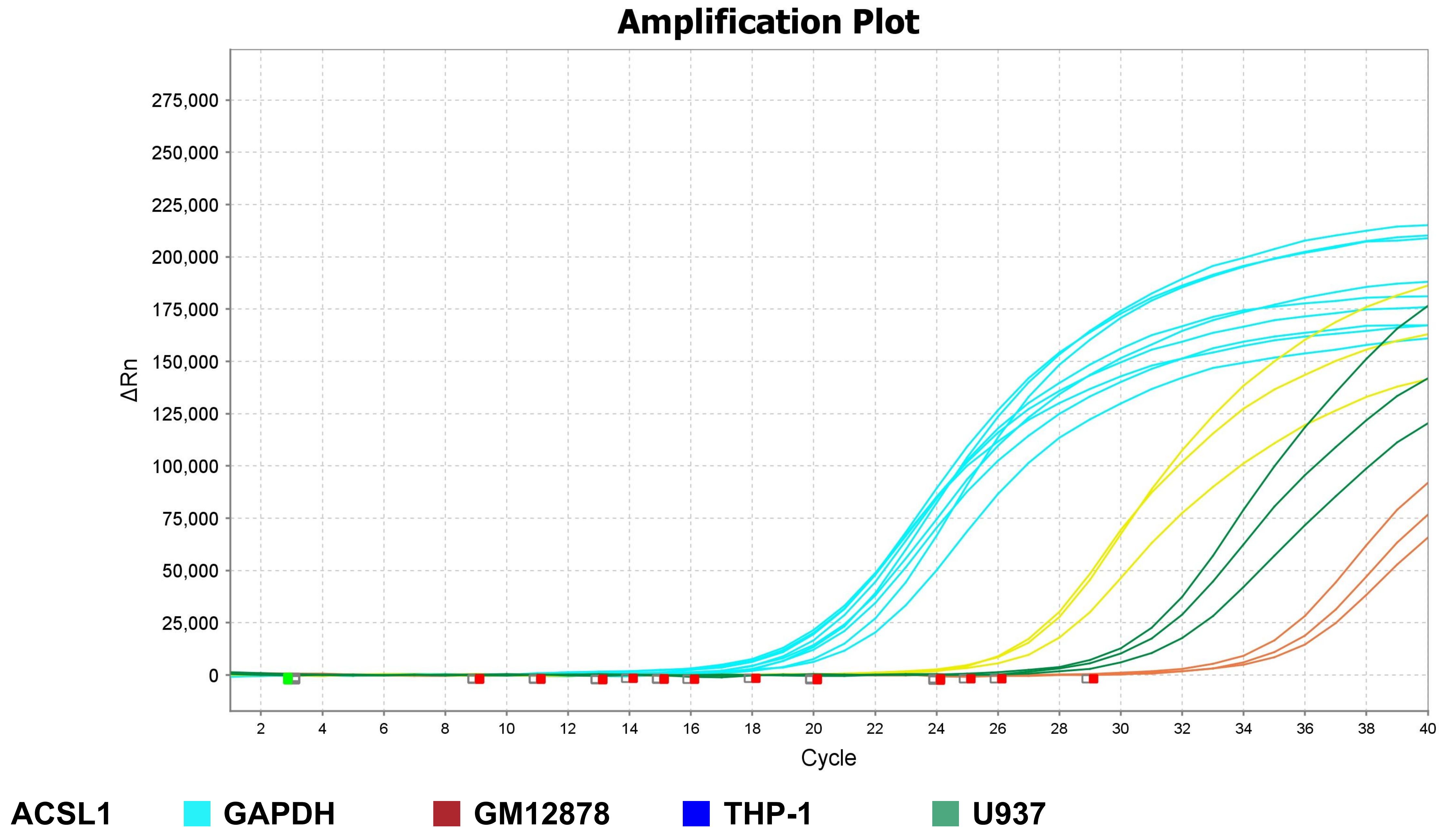

Amplification Plot
ΔRn
275,000
250,000
225,000
200,000
175,000
150,000
125,000
100,000
75,000
50,000
25,000
0
2 4 6 8 10 12 14 16 18 20 22 24 26 28 30 32 34 36 38 40
Cycle
ACSL1
GAPDH
GM12878
THP-1
U937

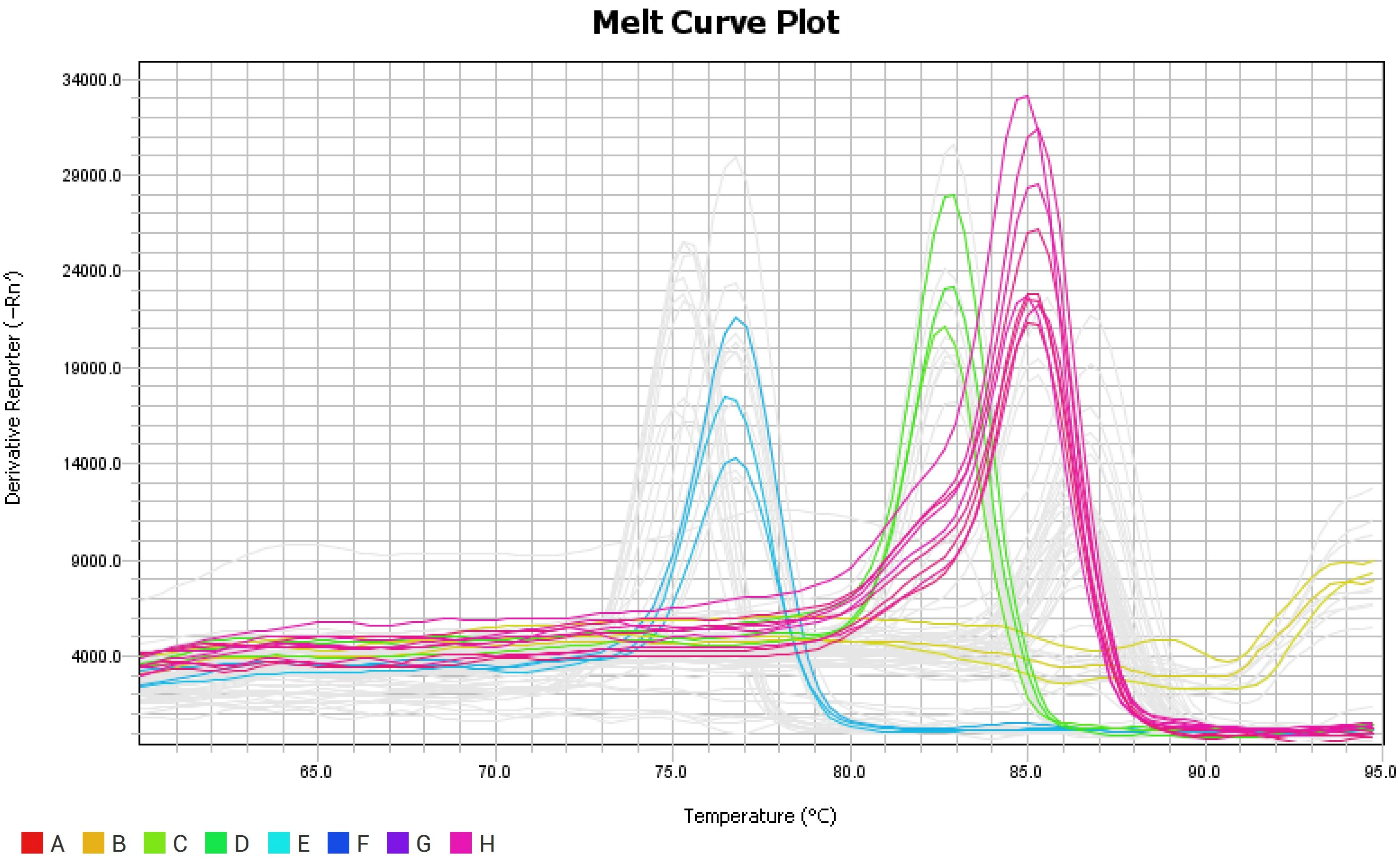

Melt Curve Plot
Derivative Reporter (−Rn')
34000.0
29000.0
24000.0
19000.0
14000.0
9000.0
4000.0
65.0
70.0
75.0
80.0
85.0
90.0
95.0
Temperature (°C)
A
B
C
D
E
F
G
H

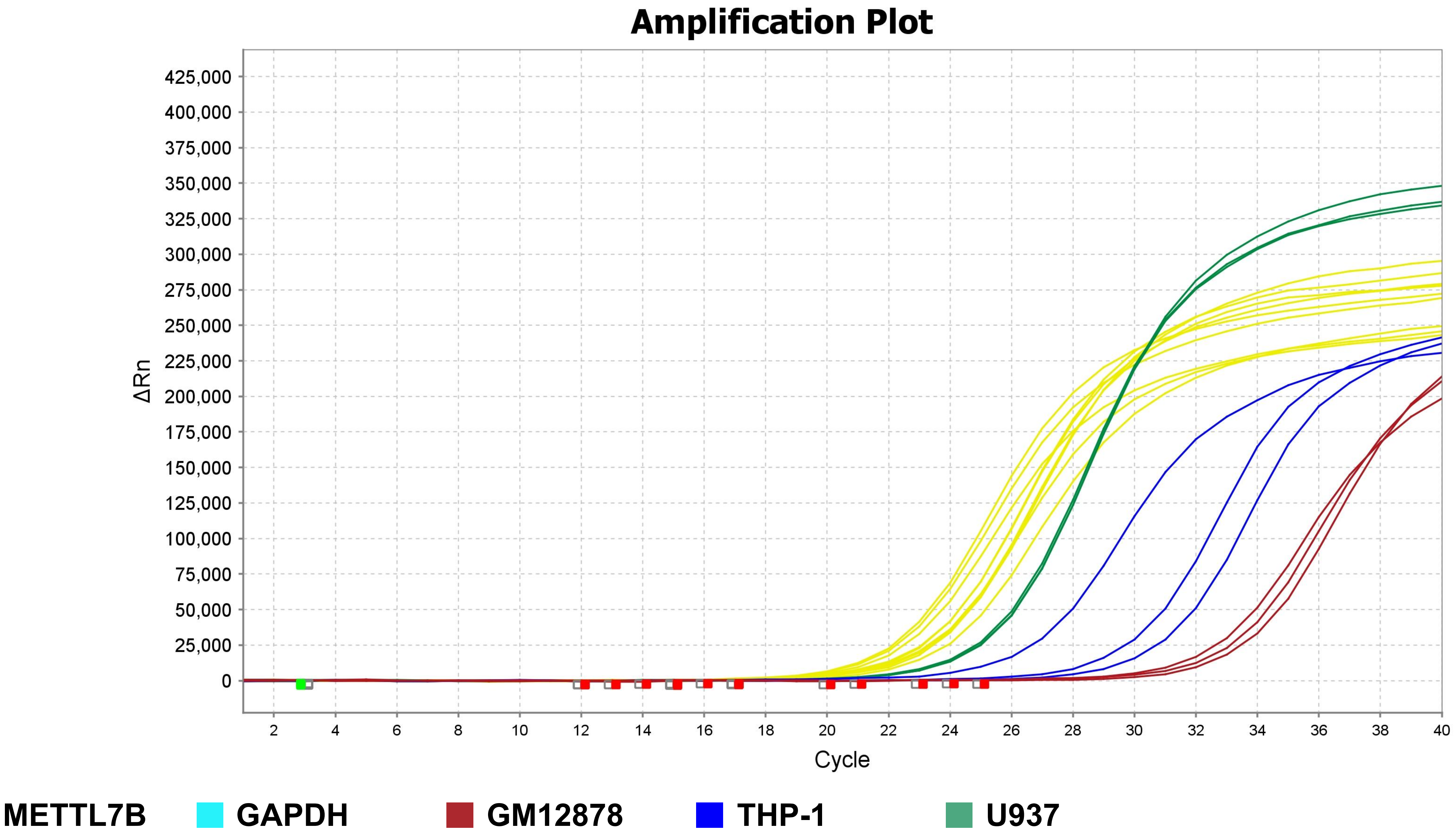

Amplification Plot
ΔRn
425,000
400,000
375,000
350,000
325,000
300,000
275,000
250,000
225,000
200,000
175,000
150,000
125,000
100,000
75,000
50,000
25,000
0
2
4
6
8
10
12
14
16
18
20
22
24
26
28
30
32
34
36
38
40
Cycle
METTL7B
GAPDH
GM12878
THP-1
U937

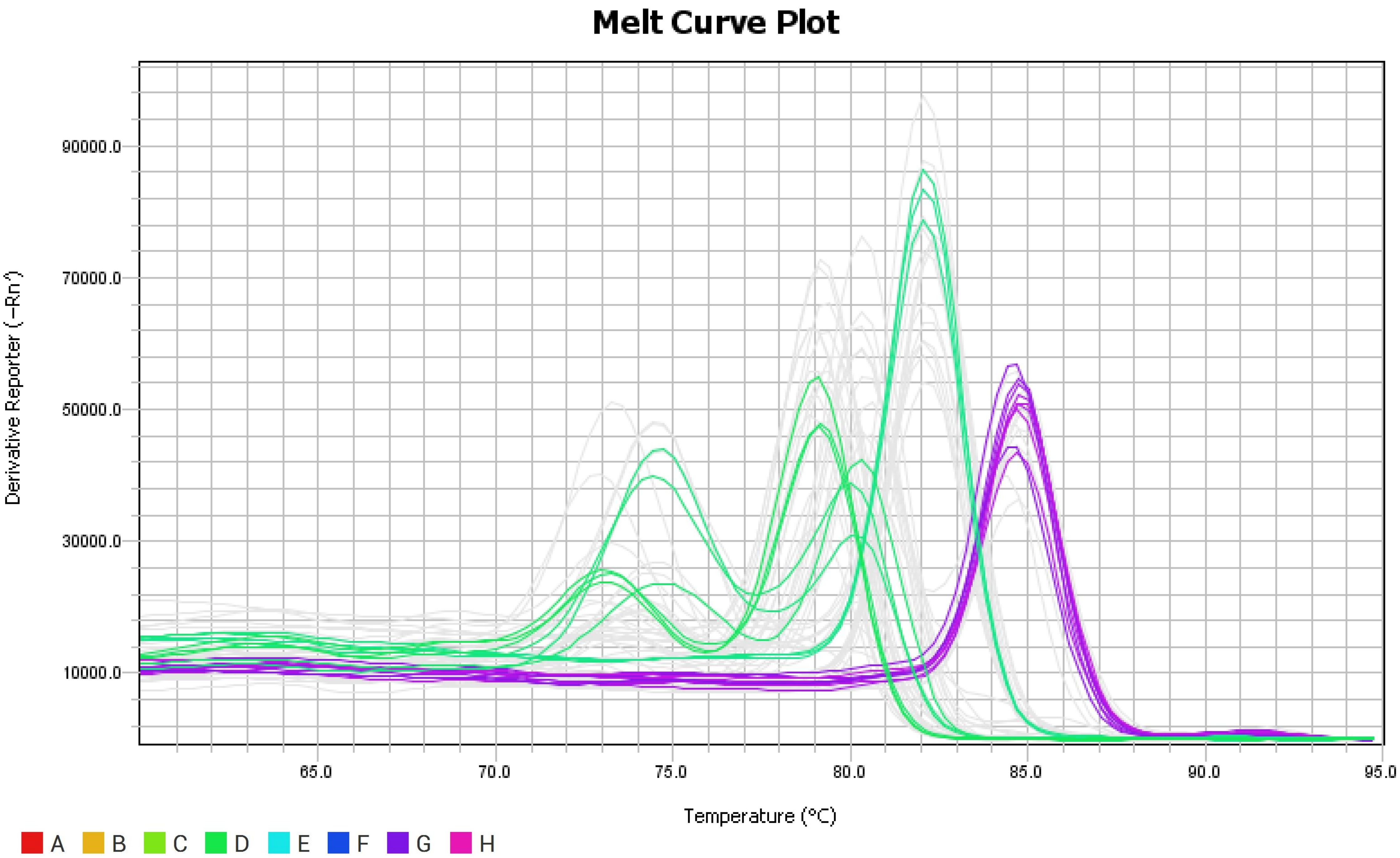

Melt Curve Plot
90000.0
70000.0
50000.0
30000.0
10000.0
Derivative Reporter (−Rn')
65.0
70.0
75.0
80.0
85.0
90.0
95.0
Temperature (°C)
A
B
C
D
E
F
G
H

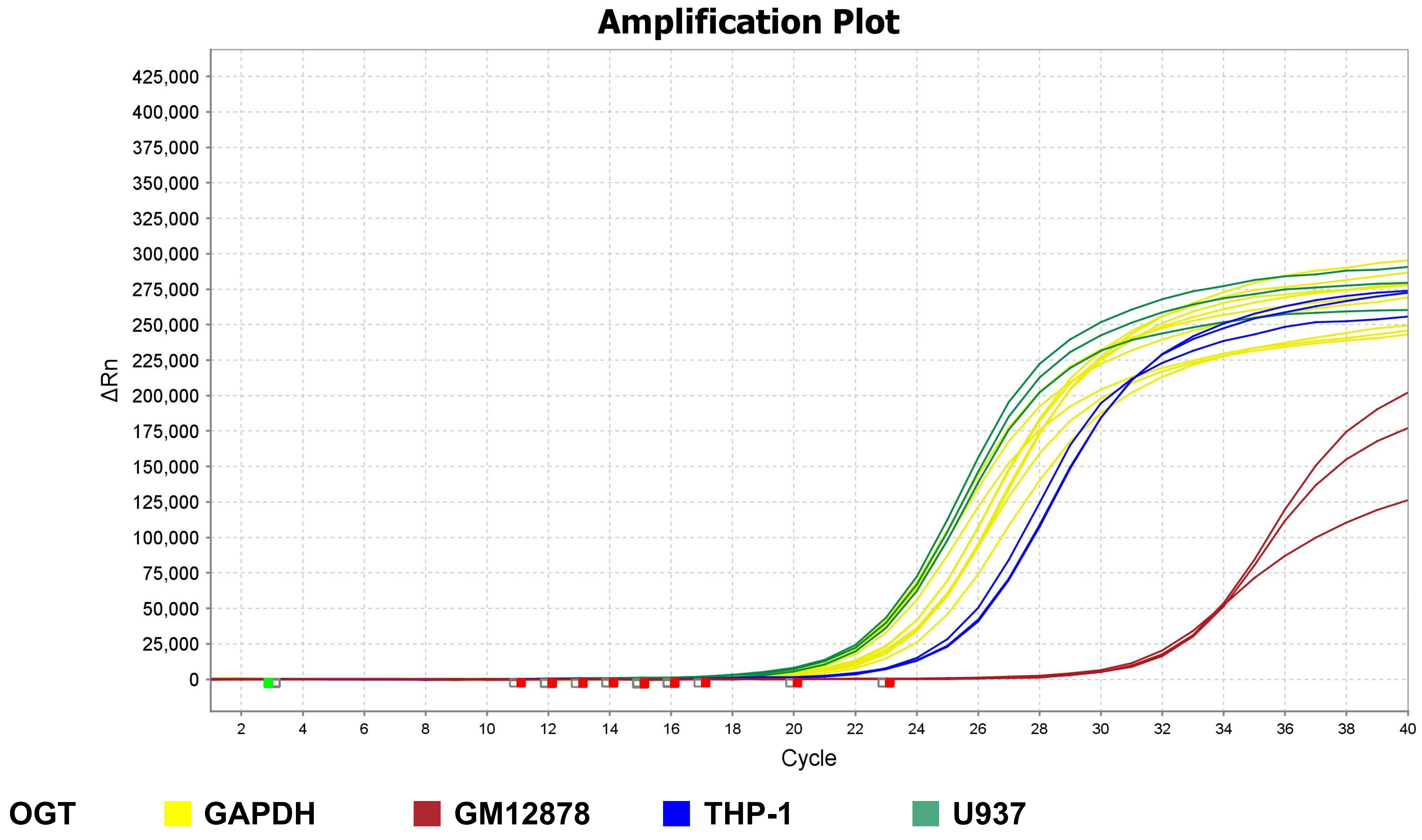
Amplification Plot
ΔRn
425,000
400,000
375,000
350,000
325,000
300,000
275,000
250,000
225,000
200,000
175,000
150,000
125,000
100,000
75,000
50,000
25,000
0
2
4
6
8
10
12
14
16
18
20
22
24
26
28
30
32
34
36
38
40
Cycle
OGT
GAPDH
GM12878
THP-1
U937

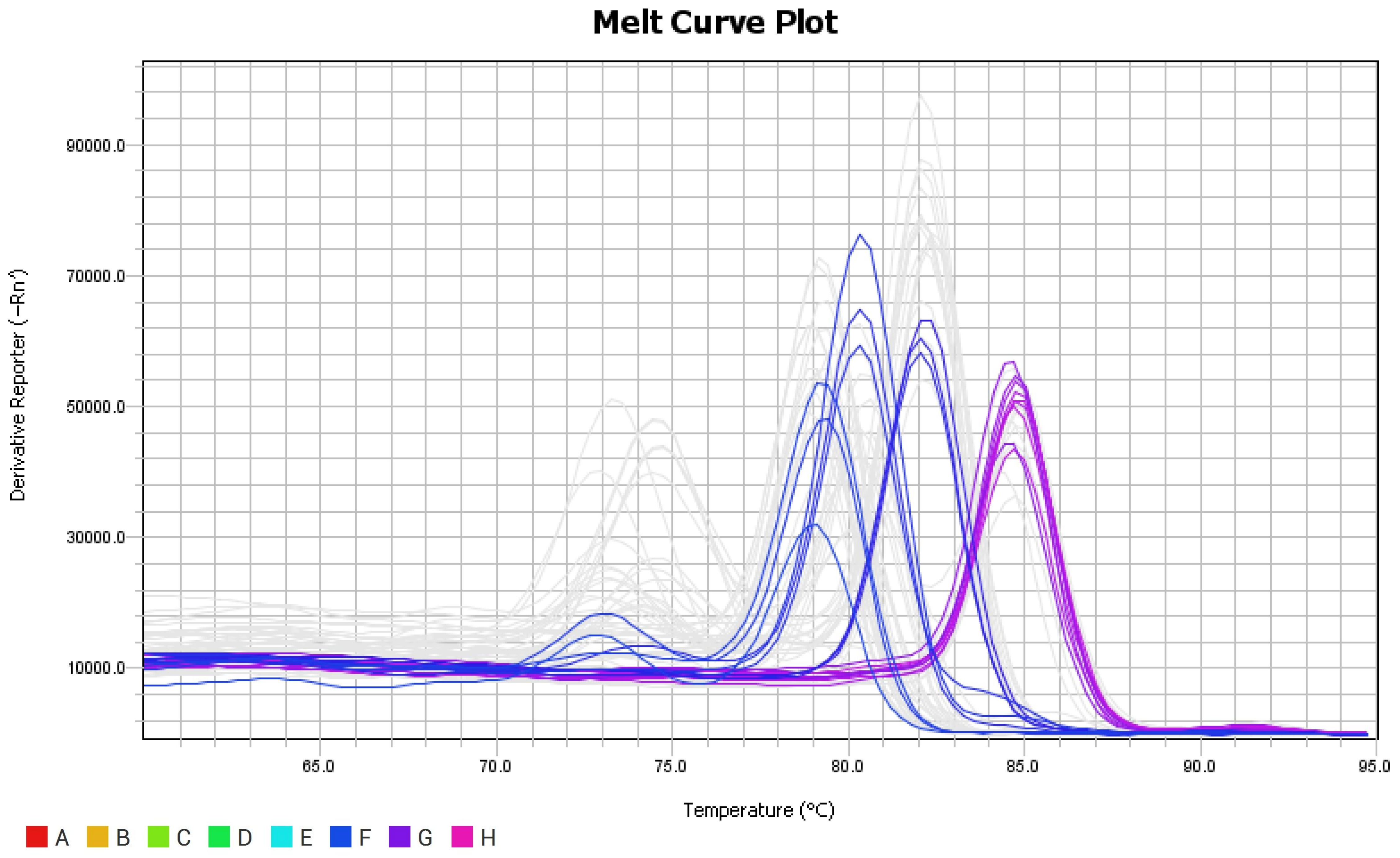
Melt Curve Plot
Derivative Reporter (−Rn')
90000.0
70000.0
50000.0
30000.0
10000.0
65.0
70.0
75.0
80.0
85.0
90.0
95.0
Temperature (°C)
A
B
C
D
E
F
G
H

Amplification Plot

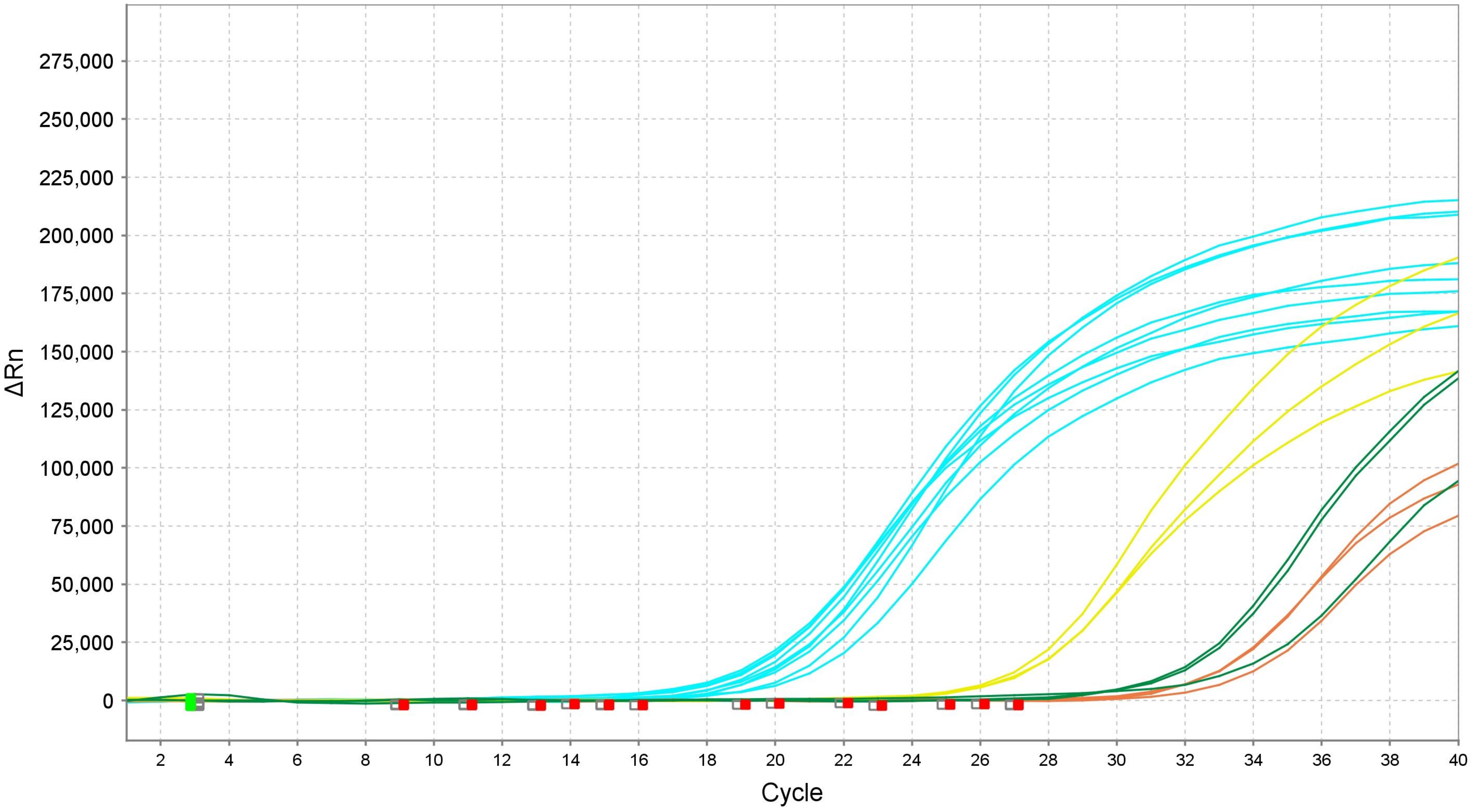

CD83 GAPDH GM12878 THP-1 U937

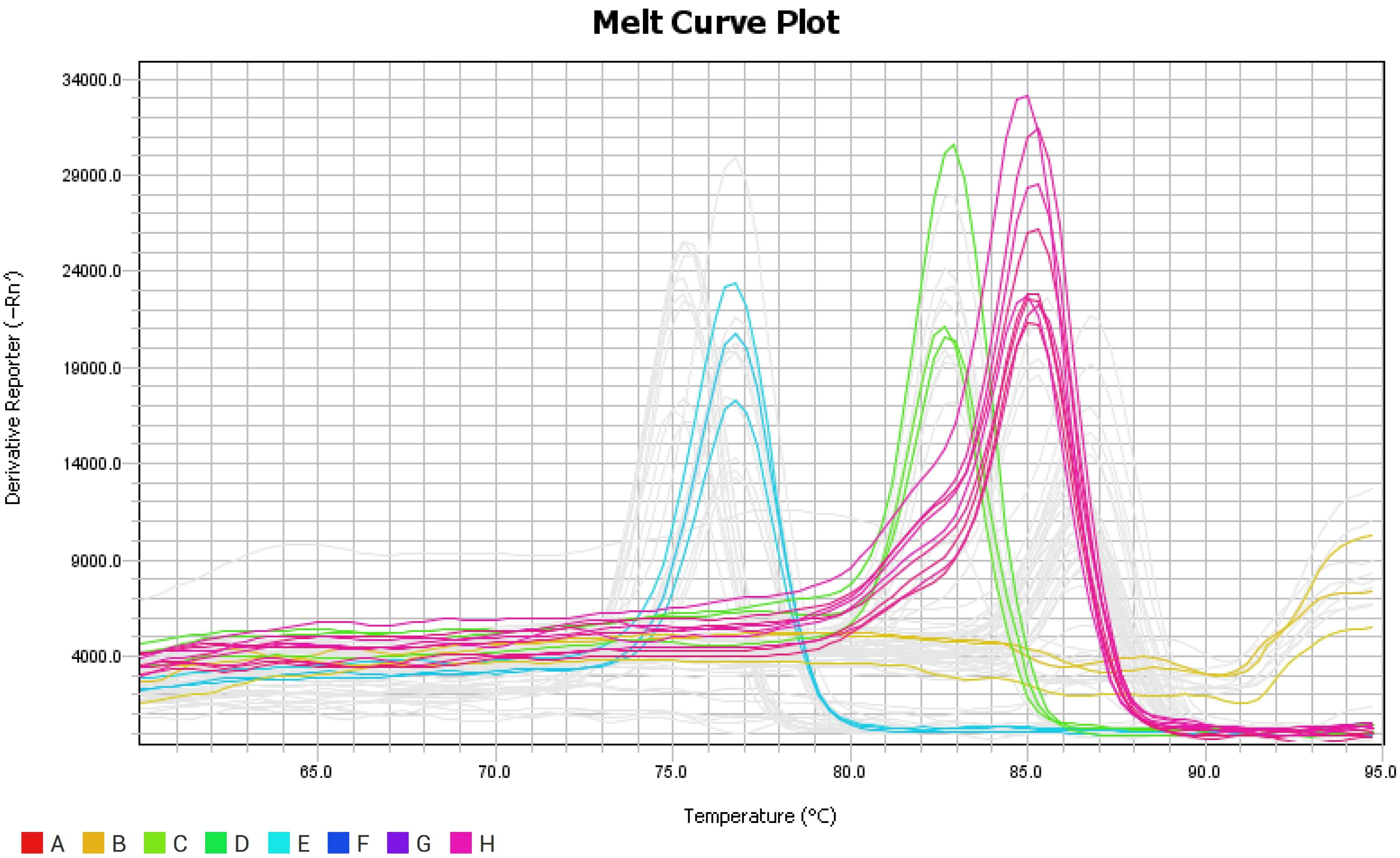

Melt Curve Plot
Derivative Reporter (−Rn')
34000.0
29000.0
24000.0
19000.0
14000.0
9000.0
4000.0
65.0
70.0
75.0
80.0
85.0
90.0
95.0
Temperature (°C)
A
B
C
D
E
F
G
H

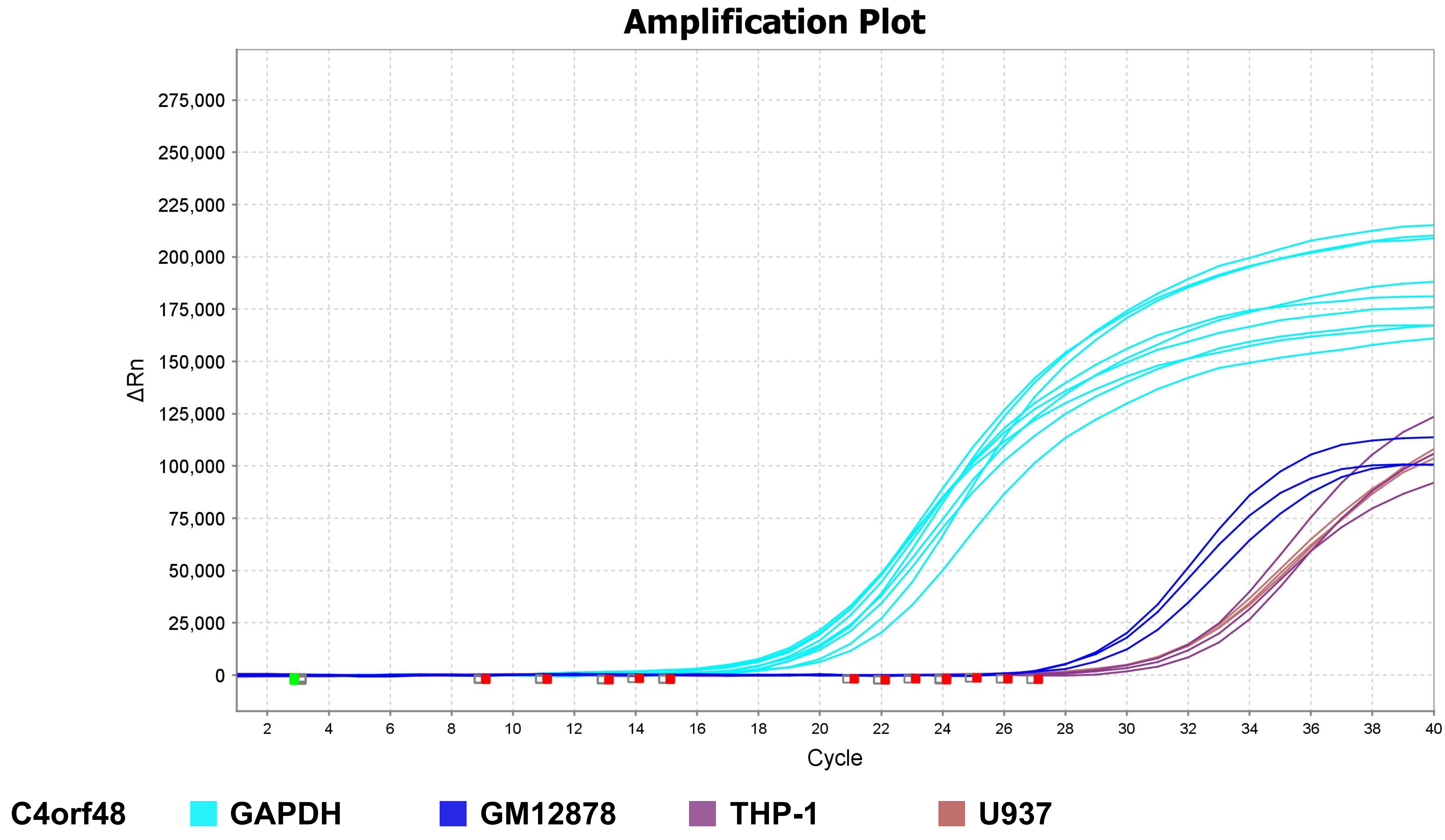

Amplification Plot
275,000
250,000
225,000
200,000
175,000
150,000
125,000
100,000
75,000
50,000
25,000
0
ΔRn
2
4
6
8
10
12
14
16
18
20
22
24
26
28
30
32
34
36
38
40
Cycle
C4orf48
GAPDH
GM12878
THP-1
U937

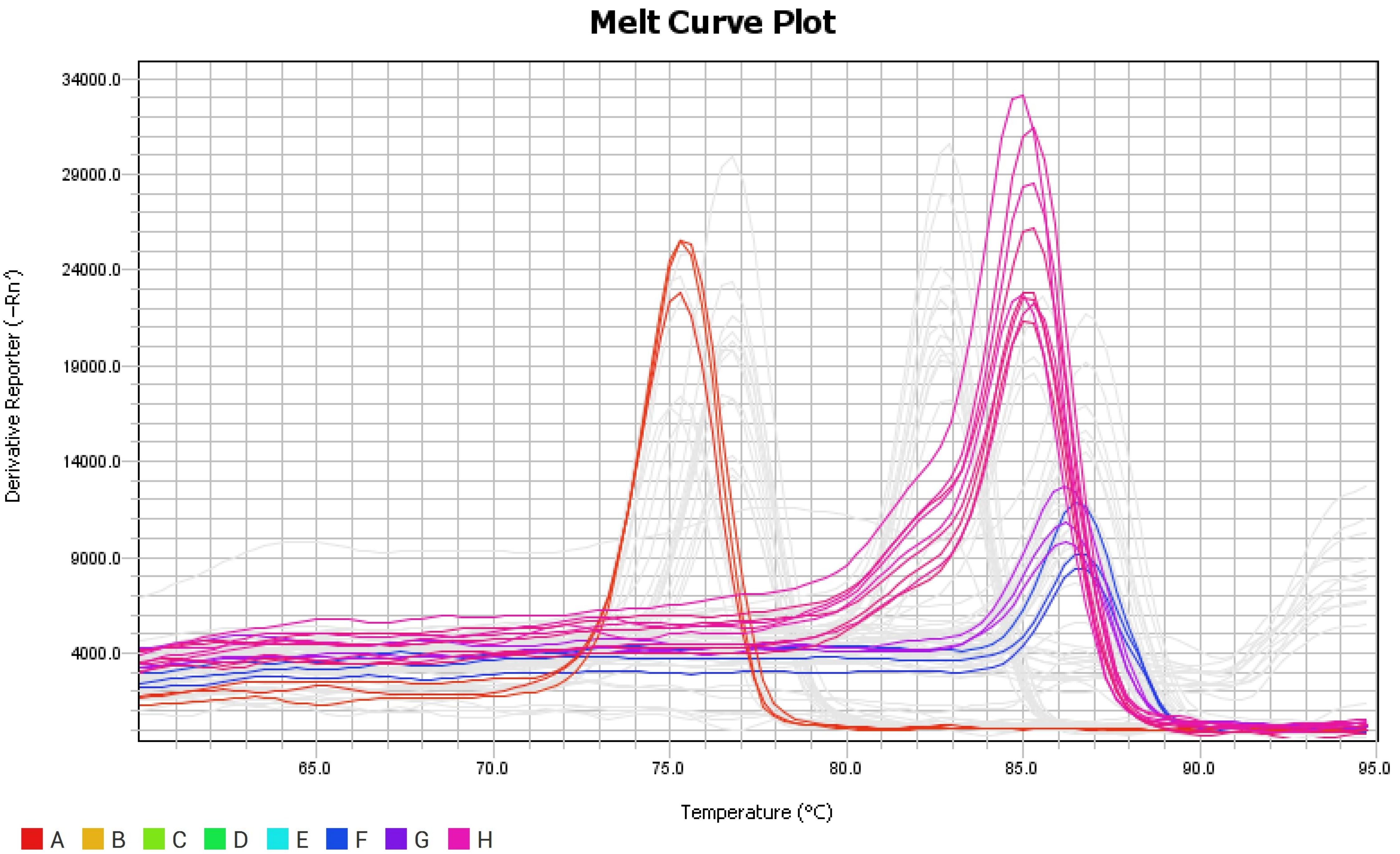

Melt Curve Plot
34000.0
29000.0
24000.0
19000.0
14000.0
9000.0
4000.0
Derivative Reporter (−Rn')
65.0
70.0
75.0
80.0
85.0
90.0
95.0
Temperature (°C)
A
B
C
D
E
F
G
H
